# Establishing a pediatric solid tumor PDX biobank for precision oncology research

**DOI:** 10.1080/15384047.2025.2541974

**Published:** 2025-08-13

**Authors:** Larissa Akemi Kido, Milena Rodrigues Marusco, Ellen Aparecida da Silva, Laís Do Carmo, Ana Beatriz Teodoro Borges, Felipe Luz Torres Silva, Juliana Silveira Ruas, Dieila Giomo de Lima, Larissa de Abreu Fernandes, Camila Maia Martin Daiggi, Izilda Aparecida Cardinalli, Mayara Ferreira Euzébio, Patricia Yoshioka Jotta, Mariana Maschietto, Priscila Pini Zenatti

**Affiliations:** a Research Center, Boldrini Children’s Center, Campinas, Brazil; b Faculty of Philosophy, Sciences and Letters at Ribeirão Preto, University of São Paulo, Ribeirão Preto, Brazil; c Boldrini Children’s Center, Campinas, Brazil; d Postgraduate Program in Genetics and Molecular Biology, Institute of Biology, University of Campinas (UNICAMP), Campinas, Brazil; e Institute of Biosciences, São Paulo State University, Botucatu, Brazil

**Keywords:** Patient-derived xenograft, solid tumors, pediatric cancer, precision medicine

## Abstract

Developing advanced preclinical models and targeted therapies is essential for reducing cancer-related deaths in children with solid tumors. Patient-derived xenografts (PDX) have the potential to replicate key elements of the original tumor, including morphology, genetic alterations, and microenvironment, making them valuable tools for studying tumor biology and drug response. We implanted 124 pediatric solid tumor samples, collected for 1 y, into NOD/SCID/IL2Rg (NSG) mice. Tumor fragments were placed subcutaneously, and the animals were monitored for up to 1 y. Histopathology, Short Tandem Repeat (STR) profiling, RT-PCR and/or RNA-sequencing were performed to confirm tumor identity and detect driver fusions. Fifty-five xenografts were successfully established (44.35% of implanted samples), representing 19 tumor types. Sarcomas, notably osteosarcoma, Ewing sarcoma, synovial sarcoma, and rhabdomyosarcoma, displayed first-generation engraftment rates above 55%. Central nervous system tumors had lower success, reflecting unique microenvironmental requirements. Histopathology and STR concordances were 85.45% and 81.1%, respectively, while 92.6% of sarcoma PDXs retained original fusion genes. Second-generation xenografts showed faster growth, suggesting adaptation to the murine host. Sporadic discrepancies, such as new fusions or lymphoproliferative expansions, indicated the need for ongoing molecular validation parallel to other techniques. A pediatric PDX biobank can effectively capture key tumor features while facilitating the study of therapeutic responses and tumor evolution. Our models confirm the feasibility of achieving stable histological and molecular profiles, offering a valuable resource for precision oncology research. Ultimately, these pediatric PDXs could accelerate the discovery of targeted therapy and significantly improve treatment outcomes.

## 1. Introduction

Patient-derived xenografts (PDXs) models consist of the xenograft of patient’s tumor sample inserted into an immunocompromised mouse, followed by histological and molecular profiling characterization^1–3^. They are widely recognized as one of the most effective models in precision medicine by supporting preclinical trials for cancer treatment.^4^ PDXs preserve the tumor’s heterogeneity, genomic alterations, gene expression profiles, and microenvironment, including all relevant cell types^2,2,5–7^ These models provide a robust platform for evidence-based decision-making in personalized therapies, aligning with the principles of precision medicine.^5^ Often referred to as patient “avatars,” they allow individualized assessment of tumor response and bridge critical gaps in translational cancer research^8,^ Gu et al., 2025).

A recent prospective study demonstrated that integrating genomic and transcriptomic profiling with high-throughput in vitro drug screening and in vivo PDX validation significantly expanded therapeutic options and improved outcomes for children with high-risk cancers.^9^ While large-scale initiatives such as MAPPYACTS^10^ have promoted precision medicine in pediatric oncology, the routine integration of functional drug testing into clinical decision-making remains limited.

In Brazil, despite advances in chemotherapy and improved outcomes, cancer remains one of the leading causes of death.^11^ According to the Brazilian National Cancer Institute (INCA), there are approximately 7,930 new cases each year.^11^ The most common pediatric cancers include leukemia (18–41%), tumors of the central nervous system (CNS) (7–17%), lymphomas (13–14%), sarcomas, renal tumors, and germ cell tumors (38–62%)^12,13^ Most of these tumors are solid, representing highly complex and heterogeneous structures.^14^ They are characterized by abnormal vasculature, often with leaky capillaries and impaired perfusion^15,16^ Many pediatric solid tumors arise from cells of the three primary germ layers.^17^ Additionally, tumor cells actively modify the surrounding stroma, creating a microenvironment that fosters their proliferation and survival.^18^ This complexity poses a significant challenge in the search for new treatment strategies, as it influences drug penetration, resistance mechanisms, and tumor progression.^19^

Although survival rates for children with solid tumors have improved with the advance of surgical procedures, chemotherapeutic regimens and radiotherapy reaching 75–80% overall survival, treatment options remain limited for high-risk, refractory, and relapsed cases.^19^ Additionally, the drugs used to treat pediatric cancer often result in long-term side effects, highlighting the urgent need for alternative therapies with reduced toxicity^20,21^ In this context, patient-derived xenografts (PDXs) emerge as a powerful preclinical model for pediatric oncology research, offering a platform to study tumor biology, test novel therapeutic strategies, and identify personalized treatment approaches^1^

From 2020 to 2023, Boldrini Children’s Hospital (Campinas, São Paulo, Brazil) attended an average of 160 pediatric patients annually diagnosed with malignant solid tumors. Since then, part of the tumor specimens has been preserved at the institutional biobank, enabling the creation of a valuable repository for research. To provide biologically relevant models and enhance the understanding of pediatric tumor biology and treatment response, we established a PDX biobank. This manuscript presents detailed data on its development, including histopathological and molecular validation, based on our single-institution experience.

## 2. Material and methods

### Sample collection

Tumor specimens were collected from pediatric patients with approval of the Research Ethics Committee of the Boldrini Children’s Hospital (CAAE: 44219021.6.0000.5376). Written informed consent for research use was obtained from the patients’ parents or legal guardians. The eligibility criteria for this study included patients aged up to 19 y old who were diagnosed with a solid tumor and underwent surgery and treatment at Boldrini Children’s Hospital. Samples were collected from 120 pediatric patients between June 2022 and August 2023. Upon arrival at the biobank, tumor tissue was washed with PBS, sectioned into small fragments, and stored at 4°C in DMEM (Dulbecco’s Modified Eagle Medium) supplemented with 1% penicillin/streptomycin. Implantation was typically performed within 2–4 h after surgical resection. This timeframe has proven sufficient to preserve tissue viability.

### PDX establishment

A total of 671 NOD/SCID/IL2Rg (NSG) immunodeficient mice (6–8 weeks old), comprising 399 females and 272 males, were utilized to establish PDX models. Food and water were provided *ad libitum*, and the animals were kept in a room with controlled temperature (25°C ±0.5), relative humidity (55% ± 1), and a 12-h light/dark cycle. Paper rolls and igloos were used for enrichment.

**Figure 1.**
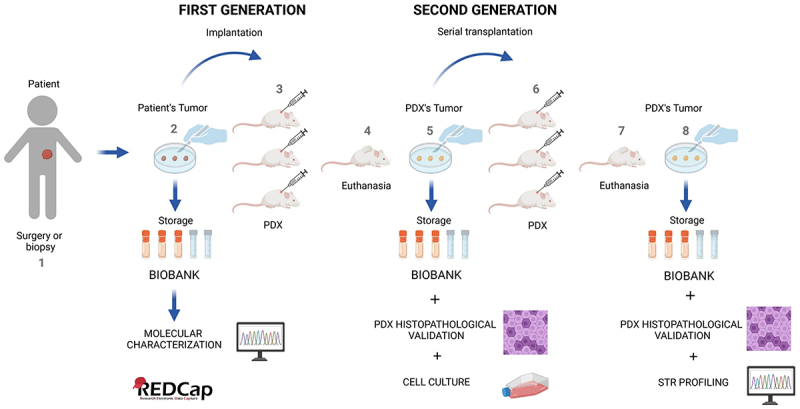
PDX establishment workflow. (1) pediatric tumor specimens were collected through surgery or biopsy at Boldrini Children’s Hospital. (2) samples were processed for biobank storage, molecular analysis, and PDX implantation, with all data registered on the RedCap platform. (3) tumor fragments measuring 5 × 5 mm were implanted subcutaneously into at least three NSG mice (1^st^ generation). (4) animals were monitored weekly for up to 12 months and euthanized when tumors reached 1,500 mm^3^ or met endpoint criteria. (5) xenograft samples were collected, stored in the biobank, validated by pathologists, and sent for cell culture analysis. (6) tumor expansion was initiated in second-generation NSG mice under the same monitoring and euthanasia criteria as the first-generation. (7) tumor samples from the second-generation were collected and processed. (8) samples were stored in the biobank, analyzed histopathologically, and validated by STR profiling. Created in BioRender. Pini Zenatti, P. (2025) https://BioRender.com/ b7kxjgu

For the implants, the mice were anesthetized intraperitoneally with 100 μL of ketamine (80 mg/kg), xylazine (10 mg/kg) and acepromazine (3 mg/kg), and placed on a heated pad at 37°C. Tumor fragments (5 × 5 mm) were embedded in a 1:1 solution of DMEM supplemented with 10% FBS and Geltrex (Thermo Fisher Scientific, Waltham, MA, USA, #Cat A1413202) and injected subcutaneously on the dorsal mouse using a needle (Cancer Implant Needle 11 g × 3–1/4” Plated Brass Hub - #Cat 7928). For the implantation of ossified tumor fragments, the mouse was positioned in ventral decubitus, and a 2 × 2 cm area of skin on the right flank was shaved. Then, a small incision (approximately 5–8 mm) was made, and the tumor fragment was placed in the subcutaneous space. The wound was then closed using a stapler, with clips removed 5 d post-implantation. These animals comprised the first-generation. The number of animals used per implant depended on the availability of tumor tissue, with a maximum of three animals of the same sex per sample (Figure 1). The experimental unit was a single animal, individually identified by ear marks (labeled A, B, and C) and had a dedicated clinical record registered in Research Electronic Data Capture (RedCaP), ensuring accurate tracking of tumor growth, procedures, and endpoint criteria throughout the experiment. Samples implanted in mice that were not diagnosed as malignant were immediately excluded from the study. In these cases, the animals were euthanized by deepening the anesthetic plane, in accordance with ethical guidelines for animal welfare.

### PDX tumor collection and biobank

Once a palpable tumor was detected, the animals were monitored weekly by weighing and measuring the tumor dimensions with calipers to calculate tumor volume using the formula V=π/6⋅length⋅width^2^. To prioritize animal welfare, the tumor size was limited to 1,000 to 1,500 mm^3^. However, humane endpoint criteria were applied in any case of signs of pain and suffering, assessed using the Grimace scale.

Animals were euthanized by lethal dose of anesthetics in association by intraperitoneal injection. The mouse was shaved in the tumor region and placed in a laminar flow hood. After asepsis of the tumor area, the skin was lifted over the tumor, and an incision was made with scissors to expose the tumor mass. At this point, photographic records were taken to identify macroscopic alterations such as necrosis, exudate, clots, among other tumor characteristics. After tumor excision, using a surgical scalpel the tumor was processed. Tumor fragments were collected for live cells biobanking, designated for histopathological analysis and stored in RNAlater® (Thermo Fisher Scientific, Waltham, MA, USA, #Cat AM7021). In cases of low tumor tissue yield (small size or presence of necrotic areas), cryopreservation and re-implantation have been prioritized over formalin fixation. To generate subsequent PDX passages, three tumor fragments measuring 5 × 5 mm were implanted into three individual mice, corresponding to the second-generation PDX. In other words, for each successful engraftment in the first-generation PDX, three new implants were performed to enable xenograft tumor expansion. Spleen, brain, heart, liver, femur, lungs and kidneys were also collected and fixed 10% neutral buffered formalin (Figure 1).

### PDX data record

Xenograft information was systematically recorded in RedCaP,^22^ a secure web-based application designed for data capture and management in research studies. Additional details are provided in Supplementary Figure S1.

### PDX validation by histopathology analysis

Formalin-fixed and paraffin embedded tumors from xenografts were sliced into 4 µm (SLEE medical GmbH – CUT 5062), stained with hematoxylin–eosin (H&E) and photographed in a Leica ICC50W microscope (Leica Microsystems, Wetzlar, Germany). The histopathological assessment and xenograft evaluation was performed by the pathologist compared to the H&E from patients’ tumors.

### Confirmation of the human origin of PDX tumor samples using Short Tandem Repeat (STR) profiling

Xenograft DNA samples were subjected to STR profiling, with a preference for tumors from second-generation PDX when available. DNA was quantified using Qubit dsDNA BR (Invitrogen™, Waltham, MA, USA, Cat#Q32853). The GenePrint24 System for STR profiling (Promega, Madison, WI, USA, Cat#B1870) was used to amplify 3 ng of template DNA in a 12.5 μL volume in a PCR, following manufacture parameters. Samples were analyzed using ABI Prism 3500 Genetic Analyzer, and STR profiles were interpreted with GeneMapper v6 (Applied Biosystems) software.

### Identification of fusion genes by RT-PCR or RNAsequencing (RNAseq)

RNA was extracted from second-generation PDX tumor samples using the RNeasy Mini Kit (Qiagen™, Hilden, Germany, Cat#74106) and complementary DNA (cDNA) was synthesized following the GoScript Reverse Transcription System protocol (Promega™, Madison, WI, USA, Cat#A5003). The quality of synthesized cDNA was verified through control PCR for commonly expressed housekeeping genes (*BCR, ABL, B2M*, and *PBGD*), ensuring sample integrity for translocation analysis.

PCR reactions were performed using GoTaq G2 polymerase (Promega™, Madison, WI, USA, Cat#M7841), specific primers for target translocations and optimized thermal cycling conditions. The translocations *SS18-SSX1*, *SS18-SSX2*, and *SS18-SSX4* were assessed for synovial sarcoma. Alveolar rhabdomyosarcoma samples were tested for *PAX3-FOXO1* and *PAX7-FOXO1* translocations, while Ewing sarcoma samples were analyzed for *EWSR1-FLI1* and *EWSR1-ERG* translocations. For Ewing sarcoma, nested PCR was employed to enhance sensitivity. All reactions were performed in duplicate or triplicate. Primer sequences and protocols are provided in (Supplementary Table S1).

RNAseq were performed as previously described.^23^ RNA samples were submitted to Illumina Stranded Total RNA Prep, Ligation with Ribo-Zero Plus (Illumina, San Diego, CA, USA) using paired-end runs. Transcriptome raw data quality was verified with FASTQC^24^ and STAR-fusion^25^ was used to align and annotate fusion transcripts based on discordant read alignments with default configurations. ChimeraViz^26^ was used to plot fusion genes. The fusion genes analyzed in this study were characterized in a cohort of 20 samples, predominantly consisting of various sarcoma types already characterized by methylation profile, including osteosarcoma, Ewing sarcoma, synovial sarcoma, rhabdomyosarcoma, clear cell sarcoma of the kidney, myeloid sarcoma and undifferentiated sarcoma.^23^

### Data analysis

Statistical analyses were performed using GraphPad Prism (version 9.0.0; GraphPad, San Diego, CA). One-way ANOVA followed by Tukey’s post hoc test was used for multiple comparisons among different tumor types, with a significance level set at 5%. For life span analysis and metastasis frequency, unpaired Student’s *t*-tests were applied. When assumptions were not met, the Mann–Whitney U test was used. Data were expressed as mean ± standard deviation.

## 3. Results

### Sample records

All information generated from PDX was stored in a customized form at REDCap (Supplementary Figure S1). Each PDX has a unique registration linked to its respective patient’s relevant clinical information ensuring patient confidentiality. Recorded data include histopathological diagnosis, birth date, gender, vital status, and tumor characteristics. Comprehensive documentation also extends to mouse-related information, such as lineage, sex, birth date, and detailed sample records. These records include sample size, implant procedure (date, responsible technician, mouse age, method used, fragment size), euthanasia data (date, responsible technician, animal weight, tumor size, tissues collected, presence of metastasis, and PDX duration), the number of tumor fragments generated, and their destination (cryopreservation, formalin fixation, or RNAlater™). After PDX registration in the REDCap web platform, the biobank management system OpenSpecimen (Krishagni Solutions Pvt. Ltd.) was used to control storage and distribution, as well as track PDX tumor specimens.

### Clinical features of pediatric patients and PDX establishment

**Figure 2.**
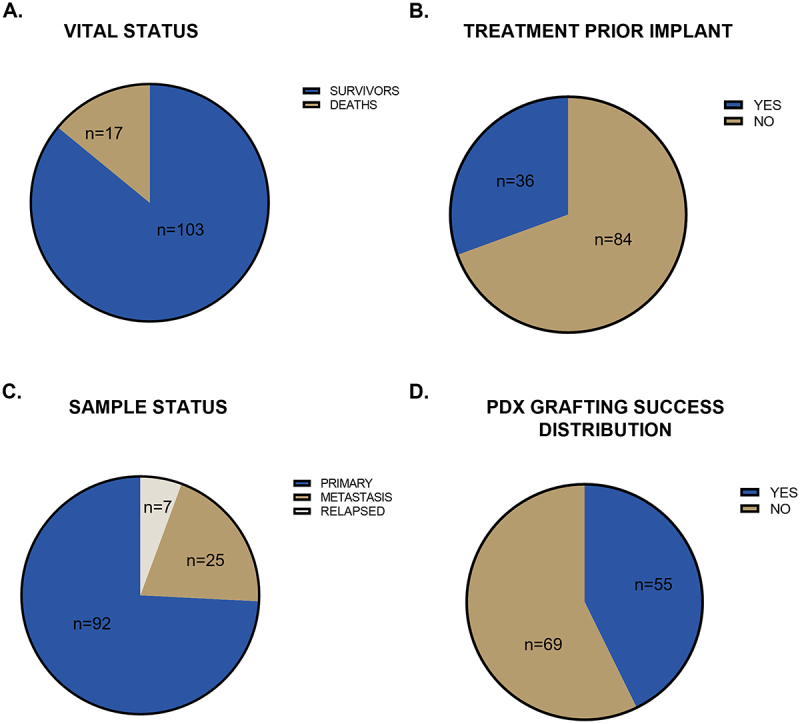
Clinical aspects of pediatric patients relevant to PDX models. (A) Patients’ vital status at the time of manuscript completion. (B) Treatment status before xenograft implantation. (C) Sample status categorized as primary site, metastasis, or relapse. (D) PDX grafting success distribution across implanted samples.

**Figure 3.**
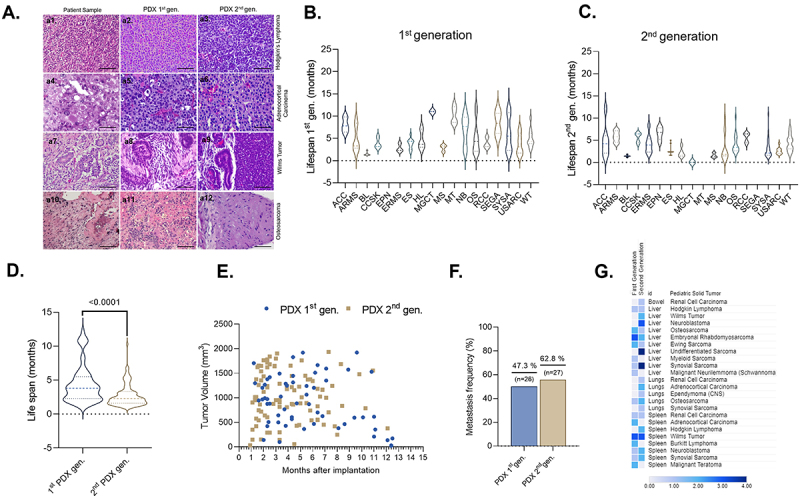
PDX main data across first and second generations. (A) Representative photomicrographs comparing morphological aspects between patient samples and corresponding PDXs. (a1-a3) Hodgkin lymphoma; (a4-a6) adrenocortical carcinoma; (a7-a9) Wilms tumor; (a10-a12) osteosarcoma. Slides stained with hematoxylin-eosin, 400× magnification, and 50 µm scale bar. (B-C) Comparison of PDX lifespan between different tumor types and passages. (D) Comparison of PDX models from first- and second-generation animals revealed a statistically significant reduction in tumor development time in the second generation (*p* < .0001). (E) Tumor growth formation after implantation. (F) Frequency of metastasis sites detected macroscopically. (G) Metastasis site frequency across different tumor types. One-way ANOVA followed by Tukey’s test and unpaired t-test, with a significance level of *p* < .05. ACC: adrenocortical tumor; ARMS: alveolar rhabdomyosarcoma; BL: Burkitt lymphoma; CCSK: clear cell sarcoma of kidney; ERMS: embryonal rhabdomyosarcoma; EPN: ependymoma; ES: Ewing’s sarcoma; HL: Hodgkin lymphoma; MGCT: mixed germ cell tumor; MT: malignant teratoma; MS: myeloid sarcoma; NB: neuroblastoma; OS: osteosarcoma: RCC: renal cell carcinoma; SEGA: subependymal glioma; SYSA: synovial sarcoma; USARC: undifferentiated sarcoma; WT: Wilms tumor.

A hundred-twenty patients were enrolled for the study that provided 124 tumor samples further implanted into NSG mice (4 patients provided samples at different time points of treatment) (Table 1). At the time of this publication, most patients were alive (Figure 2a), with a relatively balanced gender distribution (55.8% male and 44.2% female). Nearly 30% of the patients had undergone chemotherapy prior to providing samples for PDX; however, this did not affect tumor engraftment (Figure 2b). The majority of implanted samples comprised primary tumors (74.2%), followed by metastases (20.2%) and relapsed tumors (5.7%) (Figure 2c). Of the implanted samples, 44.35% (*n* = 55) successfully engrafted, while the remaining 55.65% (*n* = 69) failed to establish tumor growth in NSG mice after 1 y post-implantation (Figure 2d). To preserve the genetic profile of the original tumor and minimize costs associated with PDX maintenance, these models were propagated only up to the second-generation. Examples of cases showing concordant histology between the patient tumor and the corresponding PDX are presented in Figure 3a, illustrating the morphological fidelity preserved in selected models.

**Table 1.** Engraftment and characterization outcomes of pediatric solid tumor samples in PDX models.

		Grafting outcome first-generation			Grafting outcome second-generation				
Pediatric Solid Tumors	Patients (n)	Implants	Samples Engrafted (n)	%	Implants	Samples Engrafted (n)	%	Histology	STR profiling
								(Concordant/Discordant)	
Adrenocortical Carcinoma	7	8	2	25	2	2	100	(2/0)	(2/0)
Alveolar Rhabdomyosarcoma	3	3	3	100	3	3	100	(3/0)	(3/0)
Burkitt Lymphoma	3	3	2	66.7	2	2	100	(2/0)	(2/0)
Chondrosarcoma	1	1	0	0	-	-	-	-	-
Clear Cell Sarcoma of the Kidney	1	1	1	100	1	0	0	(1/0)	(1/0)
Desmoplastic Medulloblastoma *	1	1	0	0	-	-	-	-	-
Diffuse Low-Grade Astrocytoma*	1	1	0	0	-	-	-	-	-
Dysembryoplastic Neuroepithelial Tumor *	2	2	0	0	-	-	-	-	-
Embryonal Rhabdomyosarcoma	3	3	3	100	3	2	100	(3/1)	(3/1)
Ependymoma*	3	3	1	33.3	1	1	100	(1/0)	(1/0)
Ewing Sarcoma	7	7	4	57.1	4	3	75	(4/0)	(4/0)
Ganglioglioma*	3	3	0	0	-	-	-	-	-
Hepatoblastoma	3	3	2	66.7	1	0	0	(0/2)	(0/2)
Hodgkin Lymphoma	11	11	2	18	2	2	100	(2/0)	(2/0)
Malignant Lymphoblastic Lymphoma	1	1	0	0	-	-	-	-	-
Malignant Neurilemmoma (Schwannoma)	1	1	0	0	-	-	-	-	-
Malignant Teratoma	5	6	3	50	3	2	66.7	(2/1)	(2/1)
Mixed Germ Cell Tumor	4	4	1	25	1	0	-	(1/0)	(1/0)
Myeloid Sarcoma	1	1	1	100	1	1	100	(1/0)	(1/0)
Neuroblastoma	11	11	4	36.4	4	4	100	(3/1)	(3/1)
Osteosarcoma	12	13	8	61.5	8	8	100	(7/1)	(6/2)
Pilocytic Astrocytoma*	7	7	0	0	-	-	-	-	-
Pilomyxoid Astrocytoma *	3	3	0	0	-	-	-	-	-
Pineoblastoma	1	1	0	0	-	-	-	-	-
Renal Cell Carcinoma	1	1	1	100	1	1	100	(1/0)	(1/0)
Rhabdoid Sarcoma	1	1	0	0	-	-	-	-	-
Subependymal Glioma *	2	2	2	100	2	0	0	(1/0)	(0/1)
Synovial Sarcoma	8	9	7	77.8	7	7	100	(6/1)	(6/1)
Undifferentiated Sarcoma	2	2	2	100	2	2	100	(2/0)	(2/0)
Wilms Tumor	11	11	6	54.5	6	6	100	(5/1)	(5/1)
Total	120	124	55			46		(47/8)	(45/10)
Engraftment success rate (%)		44.35			83.64				

*Central nervous system tumors.

#Discordant samples with positive engraftment.

†Tumor types with multiple entries from the same patient.

Several tumor types demonstrated relatively high engraftment rates in the first-generation, such as synovial sarcoma (77.8%) and osteosarcoma (61.5%), with both tumor types maintaining successful engraftment into the second-generation. Similarly, alveolar rhabdomyosarcoma and embryonal rhabdomyosarcoma exhibited robust engraftment across generations (Table 1). More details are provided in the Supplementary Table S2 and Supplementary Figure S2.

Conversely, neuroblastoma and Wilms tumor displayed moderate engraftment success, with first-generation rates of 36.4% and 54.5%, respectively. Although these rates were initially lower, they achieved successful engraftment in the second generation. Tumors such as hepatoblastoma and malignant teratoma presented discordance between histological and STR profiles in some cases (Table 1).

Among CNS tumors, no engraftment was observed for pilocytic astrocytoma, diffuse low-grade astrocytoma, and desmoplastic medulloblastoma. In contrast, ependymoma achieved an engraftment rate of 33.3%, while subependymal glioma achieved 100% in the first generation (Table 1). Notably, while engrafted ependymoma samples retained both histological and STR concordance with their original tumors, subependymal glioma displayed discordant STR results (Table 1).

Differences in tumor engraftment times were observed across PDX models. For instance, the Burkitt lymphoma PDX showed rapid growth, with a mean engraftment time of 1.5 months (SD = 0.38; CV = 25.61%), whereas the malignant teratoma PDX exhibited slower progression, averaging 10.2 months (SD = 3.3; CV = 18.96%). In contrast, models such as undifferentiated sarcoma (mean = 4.7 months, SD = 3.9; CV = 66.1%) and osteosarcoma (mean = 5 months, SD = 3.6; CV = 73.80%) displayed higher intra-group variability. In the second generation, Burkitt lymphoma PDXs also demonstrated rapid growth with a mean engraftment time of 1.42 months (SD = 0.235; CV = 16.53%). Conversely, the alveolar rhabdomyosarcoma and ependymoma models exhibited slower growth, with means of 5.84 and 6.5 months, respectively. Notably, the neuroblastoma PDXs demonstrated high intra-group variability in the second generation PDX (mean = 3.20 months, SD = 3.053; CV = 95.42%) (Figure 3b,c; Supplementary Table 3).

When analyzed collectively, without distinction by tumor type, second-generation PDX animals exhibited significantly shorter survival times compared to those of the first generation (Figure 3d). This difference also reflects accelerated tumor growth, as second-generation tumors reached comparable volumes to those of the first generation in a shorter period (Figure 3e; Supplementary Figure S3). However, looking for tumor-specific groups, significant reductions in lifespan were found in Ewing sarcoma, myeloid sarcoma, synovial sarcoma, malignant teratoma, neuroblastoma, and Hodgkin lymphoma (Supplementary Figure S4).

During tumor collection, macroscopic metastases were recorded as relevant findings for the PDX models, and the most common metastatic sites were liver and spleen. Synovial sarcoma, neuroblastoma, and alveolar rhabdomyosarcoma frequently exhibit aggressive dissemination (Figure 3f,g).

### Fusion genes confirm the identity of xenograft tumors

Twenty-six bone and soft tissue sarcomas cases were submitted to RT-PCR and/or RNA-seq to unveil fusion genes. PDX tumors were considered concordant if they satisfied at least one of the following criteria: (1) The PDX harbors the same structural variant observed in the patient’s tumor; (2) The PDX exhibits a structural variant specific to the tumor type (Supplementary Table 4; Supplementary Figure 5, 6).

Regarding the gene fusion, PDX tumors showed 96.2% concordance (25 out of 26 cases) with the patient’s original tumors (Supplementary Table 4). Five sarcoma synovial samples (PDX 26, 39, 75, 102 and 161) presented the patient’s tumor driver *SS18-SSX1 translocations* (Supplementary Figure 5). However, one xenograft tumor initially classified as synovial sarcoma (PDX 40), presented the same STR profile of the original tumor but not the *SS18-SSX1* translocation (Supplementary Table 4). Moreover, the histology features of the PDX 40 did not resemble those of the patients’ tumor. The first PDX generation tumor was very necrotic with few viable areas of small round cells, differently from patient samples which were depicted by two compartments: a glandular composed by epithelioid cells arranged in a glandular pattern, and stromal component composed by fusiform cells (Supplementary Figure 7A, a1-a4). In the second PDX generation, lymphoid tissue was clearly observed along with areas of geographic necrosis (Supplementary Figure 7A, a5-a6). Three xenograft sample replicates were submitted to cytometry analysis that identified 97% of human CD45^+^ cells, confirming the development of lymphoproliferative tumors derived from human T cells (Supplementary Figure 7B). Therefore, the tumor that developed in PDX 40 originated from lymphoid tissue, likely due to missampling prior to implantation in mice, as the patient’s tumor sample was derived from a metastatic site in the cervical lymph nodes.

Another case involved a sample initially diagnosed pathologically as alveolar rhabdomyosarcoma (PDX 156), but molecular analysis reclassified it as angiomatoid fibrous histiocytoma (Supplementary Table 4). Although STR and histological analyses of the PDX were concordant, the driver fusions *PAX3-FOX1* and *PAX7-FOX1* (which are present in the majority of alveolar rhabdomyosarcoma cases) were absent in both the patient and PDX samples.

**Figure 4.**
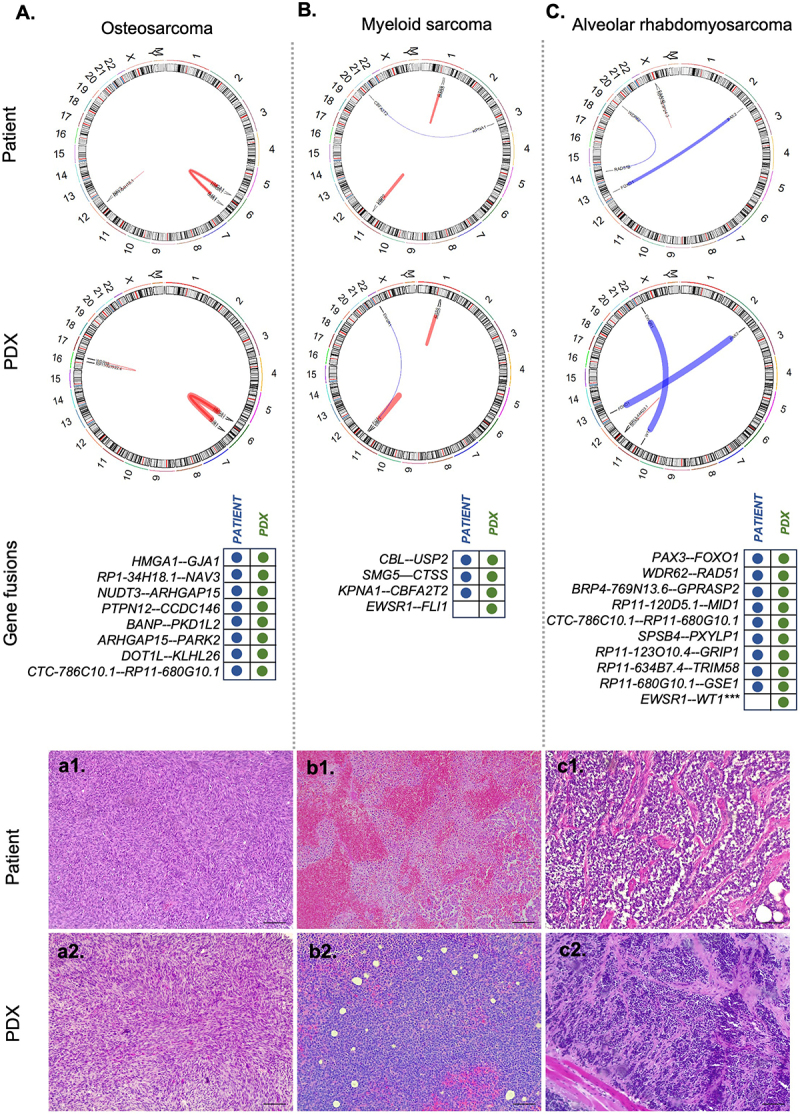
Examples of circos plots illustrating fusion genes detected by RNA sequencing in (A) osteosarcoma, (B) myeloid sarcoma, and (C) alveolar rhabdomyosarcoma, comparing patient tumors with their corresponding PDX models. Chromosomes are displayed with cytoband information, and fusion events are represented as links between chromosomal locations with corresponding gene names. Red links indicate intrachromosomal fusions, whereas blue links denote interchromosomal fusions. The width of each link reflects the number of supporting reads for the fusion event. Structural variants identified in patient tumors were also present in the corresponding PDX models, and histopathological analysis confirmed that xenograft tumors retained the key morphological features of their respective tumor types (a1-c2). slides were stained with hematoxylin and eosin, viewed at 100× magnification, with a 20 µm scale bar.

For illustration purposes, we detailed three cases (Figure 4). The PDX 67, originated from a sample of a high-grade osteosarcoma with fibroblastic morphology exhibited an STR profiling consistent with the original tumor. The xenograft also retained key morphological characteristics, including a predominance of fusiform cells with hyperchromatic nuclei of varying sizes. Additionally, all eight fusion genes identified in the original tumor were preserved in the xenograft tumor (Figure 4A, a1-a2; Supplementary Table S4). The PDX 49 represents a unique case of myeloid sarcoma within this cohort, and retained STR, morphology and fusions (*CBL – USP2, SMG5–CTSS, and KPNA1–CBFA2T2*) identical to those of the original tumor. An additional fusion, *EWSR1-FLI1*, was also detected in this PDX, which may have arisen de novo during xenograft development, or been present in the original tumor but undetected due to its occurrence in a subclonal population that was selectively expanded in the PDX. However, the presence of all patient-associated variants and similar histology, characterized by round immature cells with different sizes and scarce cytoplasm, justify its classification as a concordant case (Figure 4B, b1-b2; Supplementary Table S4). The third example involves the implantation of an alveolar rhabdomyosarcoma in mice, PDX 146, a xenograft model derived from an alveolar rhabdomyosarcoma. This model exhibits an STR profile similar to that of the patient sample and maintained the driver translocation *PAX3–FOXO1* and other additional fusion events (*WDR62-RAD51B, RP4-769N13.6-GPRASP2, RP11-120D5.1-MID1, CTC-786C10.1-RP11-680G10.1, SPSB4-PXYLP1, RP11-123O10.4-GRIP1, RP11-634B7.4-TRIM58, RP11-680G10.1-GSE1*), including a new fusion *EWSR1–WT1*. Histopathological analysis confirms the model’s fidelity characterized by the presence of small round cells with scant cytoplasm, hyperchromatic nuclei, and neoplastic cells distributed along fibrotic septa (Figure 4C, c1-c2; Supplementary Table S4)

## 4. Discussion

The establishment of a PDX biobank is critical for advancing pediatric oncology by bridging preclinical models with personalized medicine. Here, we present PDX models derived from pediatric solid tumors at a single Brazilian center, providing insights into engraftment success in over 30 tumor types. Through morphological, STR profiling and gene fusion analysis, we established 55 PDX models representing 19 distinct tumor types. Our study underscores both the complexities inherent in creating these models and their potential to further our understanding of pediatric solid tumors.

PDX tumors exhibited 85.45% and 81.1% concordance for histological and STR profiling, respectively. RNA-seq analysis confirmed that 92.6% of xenograft sarcomas carried the same fusion gene as their corresponding patient tumors. Engraftment success varied by tumor type, reflecting differences in tumor status, sample availability, and implant site. These challenges are amplified in pediatric oncology, where tumors are rarer, sample volumes are smaller, and ethical considerations limit tissue collection, making each successful PDX even more valuable. The present data showed an overall success rate of 45.5%, which is consistent with rates reported in similar studies (30–47%)^10,27,28^ Additionally, first-generation tumors required more time to reach a specific volume than second-generation tumors, suggesting that tumor cells adapt to the murine microenvironment after the initial passage, resulting in accelerated growth in subsequent generations.^10^

Among the 124 tumor samples implanted, sarcomas accounted for the highest number of successful engraftment with first-generation engraftment rates exceeding 55% for Ewing sarcoma, osteosarcoma, synovial sarcoma, and both alveolar and embryonal rhabdomyosarcoma. Previous studies reported engraftment rates ranging from 36% to 48.5% for osteosarcoma and from 24% to 41.5% for Ewing sarcoma^27,29,30^. While some studies showed engraftment rates of about 50% for rhabdomyosarcoma PDXs^30,31^ our models reached 100% successful in the first-generation.^30,31^ Interestingly, our synovial sarcoma model not only achieved an engraftment rate of 77.8% compared to the 50% previously reported but also exhibited a shorter engraftment period of 6 months compared to 10 months in the first-generation.^27^ Although variations may arise from differences in protocols, sample quality, or intrinsic factors specific to each tumor type, PDX models of pediatric sarcomas have proven their ease of expansion and adaptability to the murine microenvironment, making them an efficient model for preclinical studies. When implemented at the time of patient diagnosis, these models can help identify aggressive tumors associated with poorer prognosis^29,30^

Other tumor types, such as CNS tumors, faced significant challenges in PDX establishment. The results presented in this work and previous findings showed lower engraftment rates for CNS tumors compared to other pediatric solid tumors.^28^ The failure to engraft tumors such as pilocytic astrocytoma or diffuse low-grade astrocytoma may stem from their unique microenvironmental requirements, which are difficult to replicate using heterotopic approach. This low success rate of establishing CNS tumors may be partly due to the low tumor grade of some samples, but more importantly, to the fact that certain tumor phenotypes are regulated by gene expression induced by the specific interaction between the tumor cell and its host organ/environment^32,33^ Although subcutaneous PDXs of CNS tumors are technically simpler to administer, they fail to fully replicate the tumor microenvironment. In contrast, the use of PDX models in pediatric brain tumors has shown promising results, particularly when tumors are implanted orthotopically.^34^ This approach has proven essential for maintaining key molecular characteristics and preserving the interaction between tumor cells and the surrounding stroma, as demonstrated in models of medulloblastoma, glioblastoma, atypical teratoid rhabdoid tumor, and ependymoma.^28^ Despite these advances, the successful establishment of CNS tumor PDXs remains challenging, largely due to their lower engraftment rates compared to other pediatric solid tumors.^28^ To overcome these limitations, future strategies may include orthotopic implantation techniques, co-implantation with supportive stromal components, and the use of humanized mouse models. These approaches have the potential to improve the biological fidelity of CNS PDXs and broaden their relevance for preclinical studies.

While PDX models are invaluable for recapitulating the complexity of human tumors, their implementation presents several challenges. A significant concern is a potential alteration in tumor biology during the engraftment process, as the tumor microenvironment may undergo modifications, influencing the representation of the original tumor^35,36^ In the present study, a synovial sarcoma sample (PDX 40) exemplified the difficulties associated with metastatic tissue engraftment. Despite confirming human origin via STR analysis, the PDX did not mirror the patient’s tumor morphology. Instead, it presented a predominantly necrotic phenotype with small round cells and the second-generation PDX revealed a lymphoproliferative lesion composed of human CD45-positive T cells. The existence of T-cell PDX tumors, as seen by us, was also observed by^37^ which suggested that these might result from xenogeneic graft versus host disease (GVHD). In this context, human T cells recognize mouse tissues as foreign, triggering an immune response that contributes to lesion formation and may lead to a misunderstanding of PDX data. It was already reported by many authors that human tumors engrafted in immunodeficient mice are susceptible to the formation of lymphocytic neoplasms, most commonly EBV-associated B-cell tumors.^37–39^ Methods such as T-cell depletion before implantation^40^ or OKT-3 administration post-inoculation^41^ have been proposed to mitigate GVHD risk and could be considered in future studies to improve PDX model fidelity.

Another notable discrepancy presented here was related to a case initially diagnosed by pathology as alveolar rhabdomyosarcoma, without detection of *PAX7-FOXO1* and PAX*3-FOXO1*. Molecular analysis subsequently reclassified this tumor as angiomatoid fibrous histiocytoma. This kind of lesion is a rare, low-grade soft tissue tumor, typically affecting children and young adults, whereas rhabdomyosarcoma is a more aggressive malignancy arising from skeletal muscle cells. Despite their distinct characteristics and behaviors, both can be diagnosed through tissue biopsy, and may sometimes be confused due to overlapping histological features. This case highlights the critical importance of integrating molecular diagnostics with pathological evaluation to ensure tumor accurate classification, which is essential for guiding appropriate therapeutic strategies and prognostic assessments.

In general, PDX models retain the molecular characteristics of their human counterparts’ tumors, maintaining stable gene expression with very low variation across passages.^42^ Based on MAPPYACTS data,^10^ driver fusion genes for several sarcomas, such as *EWSR1-FLI1* in Ewing sarcoma and *PAX3-FOXO1* in rhabdomyosarcoma.^10^ Gene fusions may be used to confirm the tumor origin of the PDX, as exemplified by the presence of EWSR1-FLI1 in 13 out of 15 Ewing sarcoma PDX, and by the detection of *FUS-ERG* and *EWSR1-FEV* in one case each.^10^ The presence of new gene fusions may indicate the selection of a subclonal population by the PDX or also the presence of genomic instability leading to the emergence of new genetic alterations, including potential fusion genes.^43^ Two PDX presented additional fusions in relation to the patient’s tumors. PDX 49 (myeloid sarcoma) had *EWSR1-FLI1* and PDX 146 alveolar (rhabdomyosarcoma) had *EWSR1-WT1*, which were not identified in the original patient tumors. Independent of the mechanisms underlying the presence of additional alterations, their detection highlights the importance of continuous genomic monitoring to ensure the fidelity of these models,^43^ especially because PDX models might be used for drug screening.

A key advantage of PDX models is their ability to preserve the original tumor’s cellular heterogeneity and microenvironment, features that are often lost in cell line-derived xenograft (CDX) models^42,44–46^ This includes not only the genetic and phenotypic diversity of the patient tumor but also stromal and extracellular matrix components, such as vasculature, which are essential for maintaining a physiologically relevant tumor microenvironment^44,45^ These attributes make PDX models especially valuable for studying tumor behavior, therapeutic response, and mechanisms of resistance in a clinically relevant context^42,47^ In contrast, CDX models, derived from immortalized cell lines, are easier and faster to establish but their adaptation to *in vitro* conditions results in reduced heterogeneity and an absence of tumor microenvironment complexity.^44,48^ While CDX models are useful for high-throughput screening and basic mechanistic studies due to their reproducibility and low cost, their predictive value for clinical outcomes remains limited.^48^

Despite some limitations, such as potential clonal selection and murine stromal cell replacement during serial passaging, PDX models remain the most robust and representative in vivo systems currently available.^46^ Their capacity to retain essential features of patient tumors supports their use in translational research, biomarker discovery, and the development of personalized therapies^42,49–51^ Ongoing refinements in PDX methodologies, such as the integration of humanized models, continue to enhance their relevance and reliability. Given the scarcity of specific tumor types in pediatric populations, PDX models are indispensable for enabling robust and biologically relevant preclinical investigations.

## Conclusions

In conclusion, the establishment of a pediatric PDX biobank is crucial for advancing oncology research by providing models that closely resemble the original tumors. Our study demonstrated the successful establishment of 55 pediatric PDX models across 19 tumor types, highlighting their potential to uncover new insights regarding tumor biology, therapeutic response, and personalized medicine. While challenges such as variable engraftment success between tumor types and tumor-specific microenvironment requirements persist, our findings emphasize the utility of these models in creating high-fidelity models to test new targeted therapies. Despite minor alterations arising from extended propagation, PDX models retain their molecular fidelity and remain one of the most effective tools for cancer research. Their continued refinement and application of these models will enable further studies to perform experiments that require live cells, such as single cell RNA-sequencing, gene silencing or overexpression, and drug screening. These investigations will enable us to answer questions related to tumor origin and behavior as well as advance in the search for drugs to treat children with cancer.

## Supplementary Material

Table S4.docx

Figure legends.docx

Supplementary Figure 6.tif

Table S1.docx

Supplementary Figure 1_p2.tif

Supplementary Figure 5.tif

Supplementary Figure 2.tif

Table S3.docx

Supplementary Figure 1.tif

Table S2.docx

Supplementary Figure 5_p2.tif

Supplementary Figure 4_p2.tif

Supplementary Figure 6_p2.tif

Supplementary Figure 7.tif

Supplementary Figure 3.tif

Supplementary Methods.docx

Supplementary Figure 4.tif

## Figures and Tables

**Table:** 

ABL – *ABL1* (Abelson Murine Leukemia Viral Oncogene Homolog)
ACC – Adrenocortical Carcinoma
ANOVA – Analysis of Variance
ARMS – Alveolar Rhabdomyosarcoma
BCR – *BCR* (Breakpoint Cluster Region)
BL – Burkitt Lymphoma
CBL – *CBL* (Casitas B-lineage Lymphoma)
cDNA complementary DNA
CD45 – CD45 (Cluster of Differentiation 45)
CCSK – Clear Cell Sarcoma of the Kidney
CNS – Central Nervous System
CTSS – *CTSS* (Cathepsin S)
CV – Coefficient of Variation
DMEM – Dulbecco’s Modified Eagle Medium
DNA – Deoxyribonucleic Acid
EPN – Ependymoma
ERMS – Embryonal Rhabdomyosarcoma
ES – Ewing Sarcoma
EWSR1 – *EWSR1* (Ewing Sarcoma RNA Binding Protein 1)
FBS – Fetal Bovine Serum
FLI1 – *FLI1* (Friend Leukemia Integration 1)
FASTQC – Fast Quality Control
GVHD – Graft Versus Host Disease
HB – Hepatoblastoma
H&E – Hematoxylin and Eosin
HL – Hodgkin Lymphoma
KPNA1 – *KPNA1* (Karyopherin Subunit Alpha 1)
MGCT – Mixed Germ Cell Tumor
MS – Myeloid Sarcoma
MT – Malignant Teratoma
NB – Neuroblastoma
NSG – NOD/SCID/IL2Rg (immunodeficient mice)
OS – Osteosarcoma
PBS – Phosphate Buffered Saline
PAX3 – *PAX3* (Paired Box Gene 3)
PAX7 – *PAX7* (Paired Box Gene 7)
PCR – Polymerase Chain Reaction
PDX – Patient-Derived Xenograft
PBGD – *PBGD* (Porphobilinogen Deaminase)
RNAlater – Nucleic Acid Preservation buffer
RNA – Ribonucleic Acid
RNA-seq – RNA Sequencing
REDcap – Research Electronic Data Capture
RCC – Renal Cell Carcinoma
RT-PCR – Reverse Transcription Polymerase Chain Reaction
SCID – Severe Combined Immunodeficiency
SEGA – Subependymal Glioma
SMG5 – *SMG5* (Smg-5 Homolog, Nonsense Mediated mRNA Decay Factor)
SS18 – *SS18* (Synovial Sarcoma Translocation Gene 18)
SSX1 – *SSX1* (Synovial Sarcoma, X Breakpoint 1)
SSX2 – *SSX2* (Synovial Sarcoma, X Breakpoint 2)
SSX4 – *SSX4* (Synovial Sarcoma, X Breakpoint 4)
STR – Short Tandem Repeat
SYSA – Synovial Sarcoma
t-test – Student’s t-test
USP2 – *USP2* (Ubiquitin-Specific Peptidase 2)
USARC – Undifferentiated Sarcoma
WT – Wilms Tumor
WT1 – *WT1* (Wilms Tumor 1)

## Data Availability

The data supporting the findings of this study can be accessed in supplementary files or from the corresponding author upon reasonable request.
