# Supplementary material for: Establishing a pediatric solid tumor PDX biobank for precision oncology research
Source: Cancer Biol Ther. 2025 Aug 13;26(1):2541974. doi: 10.1080/15384047.2025.2541974 (PMC12351738; doi:10.1080/15384047.2025.2541974)
Supplement: Table S4.docx [file KCBT_A_2541974_SM8606.docx]

**Table S4.** Fusion genes validation of sarcomas xenografts tumors versus patients’ original tumors.

| PDX ID | Diagnosis | Molecular classification | Patient's structural variant | PDX's structural variant | Molecular validation |
| --- | --- | --- | --- | --- | --- |
| 9 | Undifferentiated  sarcoma | Inflammatory environment † | *RP11-384F7.2--LSAMP CTD-2547E10.3--CRYM-AS1* | *RP11-384F7.2--LSAMP CTD-2547E10.3--CRYM-AS1* | Concordant |
| 21 | Ewings sarcoma | Ewings sarcoma | N/A | *EWSR1--FLI1** | Concordant |
| 22 | Small blue round cells sarcoma | CNS *CIC* rearranged sarcoma | *CIC--NUTM1 CTC-786C10.1--RP11-680G10.1 RP11-680G10.1--GSE1 RP11-680G10.1--GSE1* | *CIC--NUTM1 CTC-786C10.1--RP11-680G10.1 RP11-680G10.1--GSE1 RP11-680G10.1--GSE1* | Concordant |
| 26 | Synovial Sarcoma | Synovial Sarcoma | SS18--SSX1 | *SS18--SSX1** | Concordant |
| 29 | Osteossarcoma | Unclassified | No important SVs | *EIF1P4--EIF1*** CCND2--CCND1**** | Concordant |
| 36 | Alveolar Rhabdomyosarcoma | Unclassified | N/A | *SMARCB1--VWF EWSR1--FLI1* | Concordant |
| 39 | Synovial Sarcoma | Synovial Sarcoma | *SS18--SSX1* | *SS18--SSX1** | Concordant |
| 40 | Synovial Sarcoma | Synovial Sarcoma | *SS18--SSX1* | No *SS18--SSX1S* translocation* | Discordant |
| 49 | Myeloid sarcoma | Inflammatory microenvironment † | *CBL--USP2 SMG5--CTSS KPNA1--CBFA2T2* | *CBL--USP2 SMG5--CTSS KPNA1--CBFA2T2 EWSR1--FLI1**** | Concordant |
| 59 | Epithelioid Cell Synovial Sarcoma | Atypical teratoid rhabdoid, subtype *MYC* | *SNHG23--MEG8 RUFY1--MAML1 SYT16--CTD-2277K2.1 CTC-786C10.1--RP11-680G10.1 RP4-756H11.3--KCTD7* | *SNHG23--MEG8 RUFY1--MAML1 SYT16--CTD-2277K2.1 CTC-786C10.1--RP11-680G10.1 RP4-756H11.3--KCTD7* | Concordant |
| 64 | Embryonal rhabdomyosarcoma | Embryonal rhabdomyosarcoma | N/A | *YAP1--BIRC2 YAP1--TMEM123* | Concordant |
| 67 | Osteosarcoma Fibroblastic | High grade osteosarcoma | *HMGA1--GJA1 RP1-34H18.1--NAV3 NUDT3--ARHGAP15 PTPN12--CCDC146 BANP--PKD1L2 ARHGAP15--PARK2 DOT1L--KLHL26 CTC-786C10.1--RP11-680G10.1* | *HMGA1--GJA1 RP1-34H18.1--NAV3 NUDT3--ARHGAP15 PTPN12--CCDC146 BANP--PKD1L2 ARHGAP15--PARK2 DOT1L--KLHL26 CTC-786C10.1--RP11-680G10.1* | Concordant |
| 75 | Synovial Sarcoma | Synovial Sarcoma | *SS18-SSX1* | SS18--SSX1* | Concordant |
| 79 | Osteossarcoma | High grade osteosarcoma | *COL5A1--RXRA BRK1--FANCD2OS EMC3--FANCD2* | *COL5A1--RXRA BRK1--FANCD2OS EMC3--FANCD2* | Concordant |
| 89 | Ewings sarcoma | Ewings sarcoma | *EWSR1--FLI1***** | No *EWSR1--FLI1* translocation* | Concordant |
| 97 | Kidney clear cell sarcoma | Kidney clear cell sarcoma | *CTC-786C10.1--RP11-680G10.1 RP11-680G10.1--GSE1 ERICH1-AS1--CTD-2281E23.2 RP11-384F7.2--LSAMP* | *CTC-786C10.1--RP11-680G10.1 RP11-680G10.1--GSE1 ERICH1-AS1--CTD-2281E23.2 RP11-384F7.2--LSAMP* | Concordant |
| 102 | Synovial Sarcoma | Synovial Sarcoma | *SS18-SSX1* | *SS18-SSX1* | Concordant |
| 107 | Synovial Sarcoma | Synovial Sarcoma | *SS18--SSX1* | *SS18--SSX1** | Concordant |
| 111 | Osteoblastic osteosarcoma | Inflammatory environment † | *BIRC6--MAP4K3 ROCK2--THADA CTC-786C10.1--RP11-680G10.1 RRP1B--MYB SMUG1--CDK4 VPS9D1-AS1--DEF8 CEP68--IGH@-ext CDK14--HGF MAP4K3--BIRC6 RP1-34H18.1--NAV3 LTBP1--GPR75-ASB3 GLO1--AGPAT3 RP11-380O24.1--SRGAP3* | *BIRC6--MAP4K3 ROCK2--THADA CTC-786C10.1--RP11-680G10.1 RRP1B--MYB SMUG1--CDK4 VPS9D1-AS1--DEF8 CEP68--IGH@-ext CDK14--HGF MAP4K3--BIRC6 RP1-34H18.1--NAV3 LTBP1--GPR75-ASB3 GLO1--AGPAT3 RP11-380O24.1--SRGAP3* | Concordant |
| 118 | Osteoblastic osteosarcoma | High grade osteosarcoma | *RP11-444D3.1--SOX5* | *RP11-444D3.1--SOX5* | Concordant |
| 129 | Ewings sarcoma | Unclassified | *RP11-662M24.2--RIMBP2 EWSR1--FLI1 RP11-380O24.1--SRGAP3 RP4-769N13.6--GPRASP2 EWSR1--FLI1* | *RP11-662M24.2--RIMBP2 EWSR1--FLI1 RP11-380O24.1--SRGAP3 RP4-769N13.6--GPRASP2 EWSR1--FLI1* | Concordant |
| 131 | Ewings sarcoma | Ewings sarcoma | *RP1-34H18.1--NAV3 MAF--RP11-679B19.2 LL0XNC01-237H1.2--LINC00630 RP4-535B20.1--JAK1 TPTE2--MRPS31P2 RP11-444D3.1--SOX5 RP11-680G10.1--GSE1 NRIP1--AF127936.7 CTC-786C10.1--RP11-680G10.1* | *EWSR1--FLI1**** | Concordant |
| 136 | Embryonal rhabdomyosarcoma | Inflammatory microenvironment † | CTC-786C10.1--RP11-680G10.1 | *CTC-786C10.1--RP11-680G10.1* | Concordant |
| 146 | Alveolar rhabdomyosarcoma | Rhabdomyosarcoma, alveolar subtype | *PAX3--FOXO1 WDR62--RAD51B RP4-769N13.6--GPRASP2 RP11-120D5.1--MID1 CTC-786C10.1--RP11-680G10.1 SPSB4--PXYLP1 RP11-123O10.4--GRIP1 RP11-634B7.4--TRIM58 RP11-680G10.1--GSE1* | *PAX3--FOXO1 WDR62--RAD51B RP4-769N13.6--GPRASP2 RP11-120D5.1--MID1 CTC-786C10.1--RP11-680G10.1 SPSB4--PXYLP1 RP11-123O10.4--GRIP1 RP11-634B7.4--TRIM58 RP11-680G10.1--GSE1 EWSR1--WT1**** | Concordant |
| 156 | Alveolar Rhabdomyosarcoma | Angiomatoid fibrous histiocytoma | *NIPBL--C5orf42 PTPRG--FHIT RP11-123O10.4--GRIP1 CTC-786C10.1--RP11-680G10.1 SPAG9--TOB1 AGFG1--TRAF3IP1 PLCE1--ATE1* | *NIPBL--C5orf42 PTPRG--FHIT RP11-123O10.4--GRIP1 CTC-786C10.1--RP11-680G10.1 SPAG9--TOB1 AGFG1--TRAF3IP1 PLCE1--ATE1* | Concordant |
| The concordant cases satisfied at least one of the following criteria: 1) PDX harbors the same fusions observed in the patient; 2) PDX exhibits fusions characteristic of the tumor type in question | | | | | |
| † Inflammatory microenvironment and unclassified means that an insuficient number of tumor cells were presented in the sample assayed for DNA methylation unabling the classifier to indicate a molecular class with calibrate score >0.9, according to https://pubmed.ncbi.nlm.nih.gov/33479225/ | | | | | |
| *** Variant found only in PDX RNA-seq | | | | | |
| ****Validated through cytogenetic analysis | | | | | |
| CNS - central nervous system | | | | | |
