# Supplementary material for: Establishing a pediatric solid tumor PDX biobank for precision oncology research
Source: Cancer Biol Ther. 2025 Aug 13;26(1):2541974. doi: 10.1080/15384047.2025.2541974 (PMC12351738; doi:10.1080/15384047.2025.2541974)
Supplement: Figure legends.docx [file KCBT_A_2541974_SM8605.docx]

**Figure legends (main manuscript)**

**Figure 1.** PDX establishment workflow. (1) Pediatric tumor specimens were collected through surgery or biopsy at Boldrini Children’s Hospital. (2) Samples were processed for biobank storage, molecular analysis, and PDX implantation, with all data registered on the RedCap platform. (3) Tumor fragments measuring 5 x 5 mm were implanted subcutaneously into at least three NSG mice (1^st^ generation). (4) Animals were monitored weekly for up to 12 months and euthanized when tumors reached 1,500 mm³ or met endpoint criteria. (5) Xenograft samples were collected, stored in the biobank, validated by pathologists, and sent for cell culture analysis. (6) Tumor expansion was initiated in second-generation NSG mice under the same monitoring and euthanasia criteria as the first-generation. (7) Tumor samples from the second-generation were collected and processed. (8) Samples were stored in the biobank, analyzed histopathologically, and validated by STR profiling.

**Figure 2.** Clinical aspects of pediatric patients relevant to PDX Models. **(A)** Patients' vital status at the time of manuscript completion. **(B)** Treatment status before xenograft implantation. **(C)** Sample status categorized as primary site, metastasis, or relapse. **(D)** PDX grafting success distribution across implanted samples.

**Figure 3.** PDX main data across first and second generations. **(A)** Representative photomicrographs comparing morphological aspects between patient samples and corresponding PDXs. **(a1-a3)** Hodgkin lymphoma; **(a4-a6)** adrenocortical carcinoma; **(a7-a9)** Wilms tumor; **(a10-a12)** osteosarcoma. Slides stained with hematoxylin-eosin, 400× magnification, and 50 µm scale bar. **(B-C)** Comparison of PDX lifespan between different tumor types and passages. **(D)** Comparison of PDX models from first- and second-generation animals revealed a statistically significant reduction in tumor development time in the second generation (p<0.0001). **(E)** Tumor growth formation after implantation. **(F)** Frequency of metastasis sites detected macroscopically. **(G)** Metastasis site frequency across different tumor types. One-way ANOVA followed by Tukey's test and unpaired t-test, with a significance level of p < 0.05. ACC: adrenocortical tumor; ARMS: alveolar rhabdomyosarcoma; BL: Burkitt lymphoma; CCSK: clear cell sarcoma of kidney; ERMS: embryonal rhabdomyosarcoma; EPN: ependymoma;  ES: Ewing’s sarcoma; HL: Hodgkin lymphoma; MGCT: mixed germ cell tumor; MT: malignant teratoma; MS: myeloid sarcoma; NB: neuroblastoma; OS: osteosarcoma: RCC: renal cell carcinoma; SEGA: subependymal glioma; SYSA: synovial sarcoma; USARC: undifferentiated sarcoma; WT: Wilms tumor.

**Figure 4.** Examples of circos plots illustrating fusion genes detected by RNA sequencing in **(A)** osteosarcoma, **(B)** myeloid sarcoma, and **(C)** alveolar rhabdomyosarcoma, comparing patient tumors with their corresponding PDX models. Chromosomes are displayed with cytoband information, and fusion events are represented as links between chromosomal locations with corresponding gene names. Red links indicate intrachromosomal fusions, whereas blue links denote interchromosomal fusions. The width of each link reflects the number of supporting reads for the fusion event. Structural variants identified in patient tumors were also present in the corresponding PDX models, and histopathological analysis confirmed that xenograft tumors retained the key morphological features of their respective tumor types **(a1-c2).**  Slides were stained with hematoxylin and eosin, viewed at 100× magnification, with a 20 µm scale bar.

**Supplementary Figure Legends**

**Supplementary Figure 1.** Figure S1. Customized electronic form for patient samples and PDX data created in the REDCap platform.

**Supplementary Figure 2.** Figure S2. Illustrative examples of short tandem repeat (STR) analysis for concordant *versus* discordant cases. Representing a concordant case (A-C) originating from adrenocortical carcinoma.

**Supplementary Figure 3. Figure S3.** Swimmer plot shows the relationship between tumor volume and engraftment duration for first- and second-generation PDX models. Each horizontal bar represents an individual PDX, with length corresponding to the time (in months) from implantation to euthanasia. The color of each bar reflects the final tumor volume reached (in mm³), as indicated by the color scale. Models from the first generation are shown in the lower (blue-shaded) while second-generation models are displayed above (beige-shaded). This visualization highlights differences in tumor take time and growth behavior between the PDX generations.

**Supplementary Figure 4.** Figure S4. PDX life span across different types of solid tumor. The results were expressed as mean ± SD. Statistical significance indicated as *p < 0.05, **p < 0.01, ***p < 0.001, ****p <0.0001. SD, standard deviation.

**Supplementary Figure 5.** Figure S5. PCR amplification of specific translocations in tumor xenograft samples. (A) Synovial sarcoma *(SS18-SSX*), (B) Ewing sarcoma (*EWSR1-FLI1/EWSR1-ERG*), and (C) Rhabdomyosarcoma (*PAX3-FOXO1/PAX7-FOXO1*). Amplified products were separated by agarose gel electrophoresis and visualized using SYBR Safe staining. L: 100 bp molecular weight ladder. Samples and controls position are described in the figure.

**Supplementary Figure 6.** Figure S6. Circos plots illustrate fusion genes detected by RNA sequencing in patients’ samples (A-Q). Chromosomes are displayed with cytoband information, and fusion events are represented as links between chromosomal locations with corresponding gene names. Red links indicate intrachromosomal fusions, while blue links represent interchromosomal fusions. The varying widths of the links correspond to the quantity of supporting reads for each fusion event. Chromosomal locations and gene names are also included. ARMS – Alveolar Rhabdomyosarcoma; CCSK – Clear Cell Sarcoma of the Kidney; ERMS – Embryonal Rhabdomyosarcoma; ES – Ewing Sarcoma; MS – Myeloid Sarcoma; OS – Osteosarcoma; SYSA – Synovial Sarcoma; USARC – Undifferentiated Sarcoma.

**Supplementary Figure 7.** Figure S7 (pdf). Example of a synovial sarcoma case in which the patient harbors *the SS18--SSX1* structural variant, but the corresponding PDX does not. The patient's tumor exhibits the characteristic biphasic histology of synovial sarcoma, with distinct stromal and epithelial components (a1, a2). In contrast, first- and second-generation PDX tumors (a3–a6) show extensive necrotic areas (asterisk) indicative of geographic necrosis, along with small, round cells with scant cytoplasm resembling lymphocytes (arrow). (B) Flow cytometry analysis of hCD45 expression (upper panel) within hCD3+/hCD20+ cells (lower panel) confirms the presence of a human lymphoproliferative xenograft tumor.
