# Supplementary figures and images for: Establishing a pediatric solid tumor PDX biobank for precision oncology research
Source: Cancer Biol Ther. 2025 Aug 13;26(1):2541974. doi: 10.1080/15384047.2025.2541974 (PMC12351738; doi:10.1080/15384047.2025.2541974)

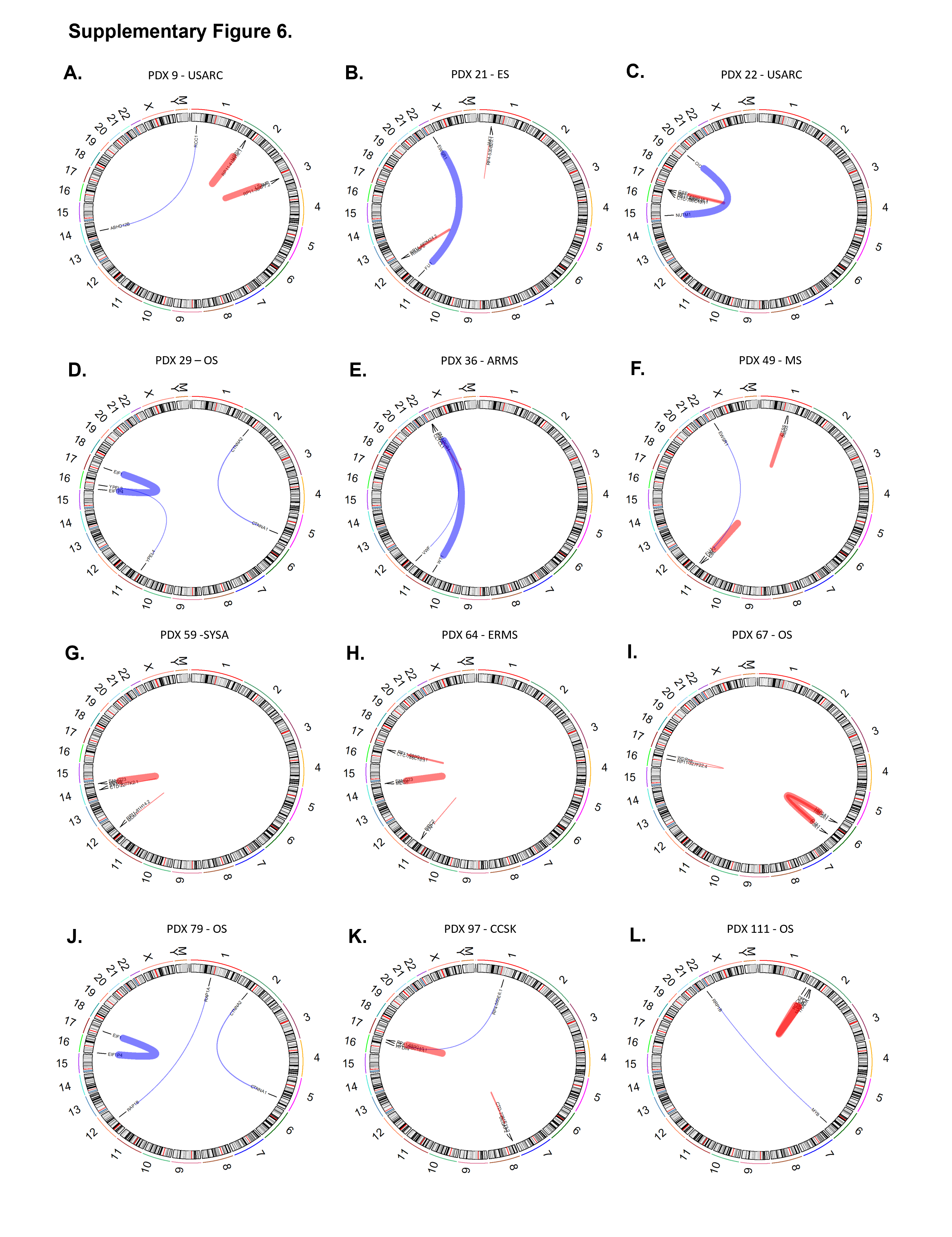

Supplement: Supplementary Figure 6.tif [file KCBT_A_2541974_SM8604.tif]

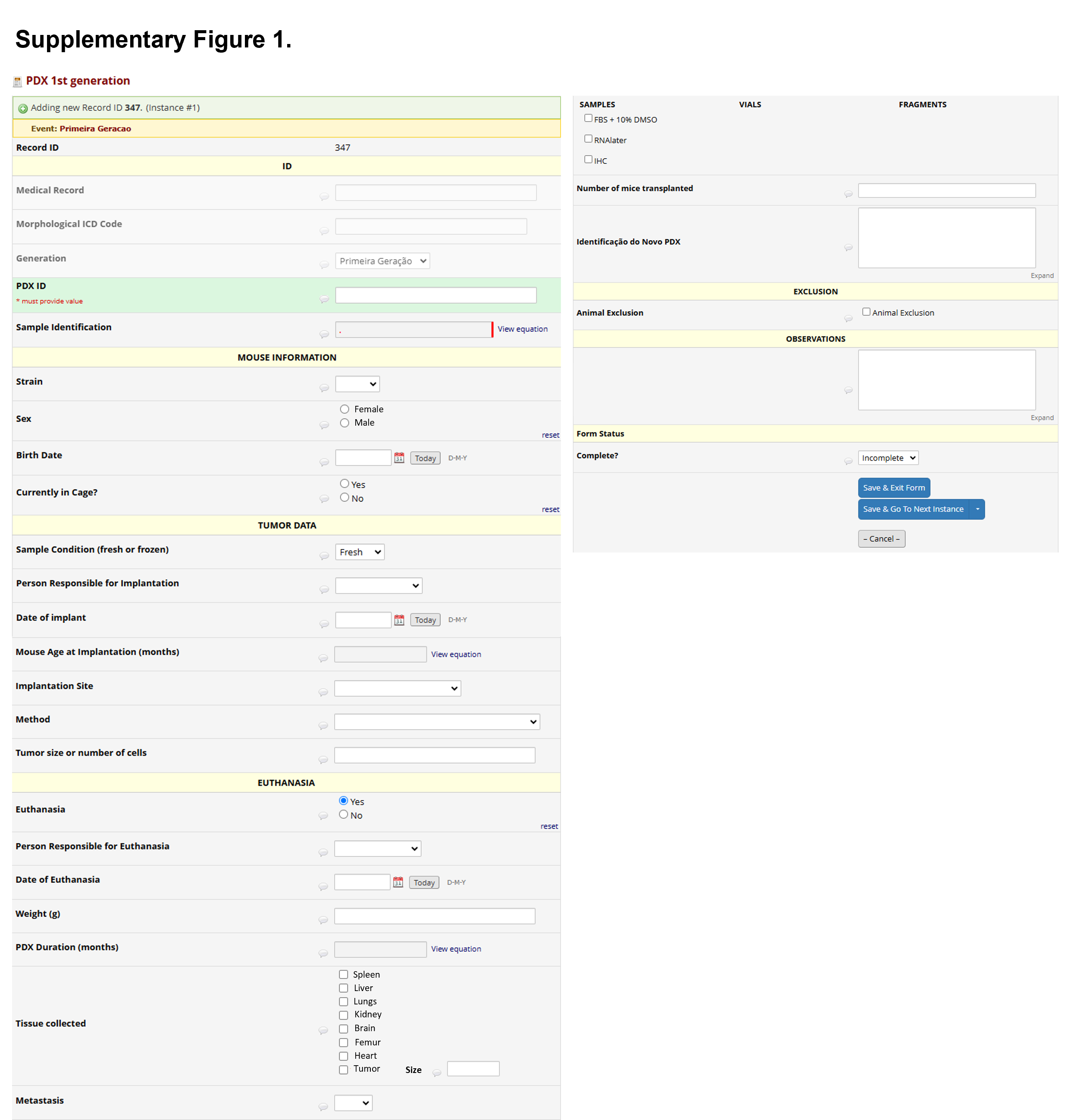

Supplement: Supplementary Figure 1_p2.tif [file KCBT_A_2541974_SM8602.tif]

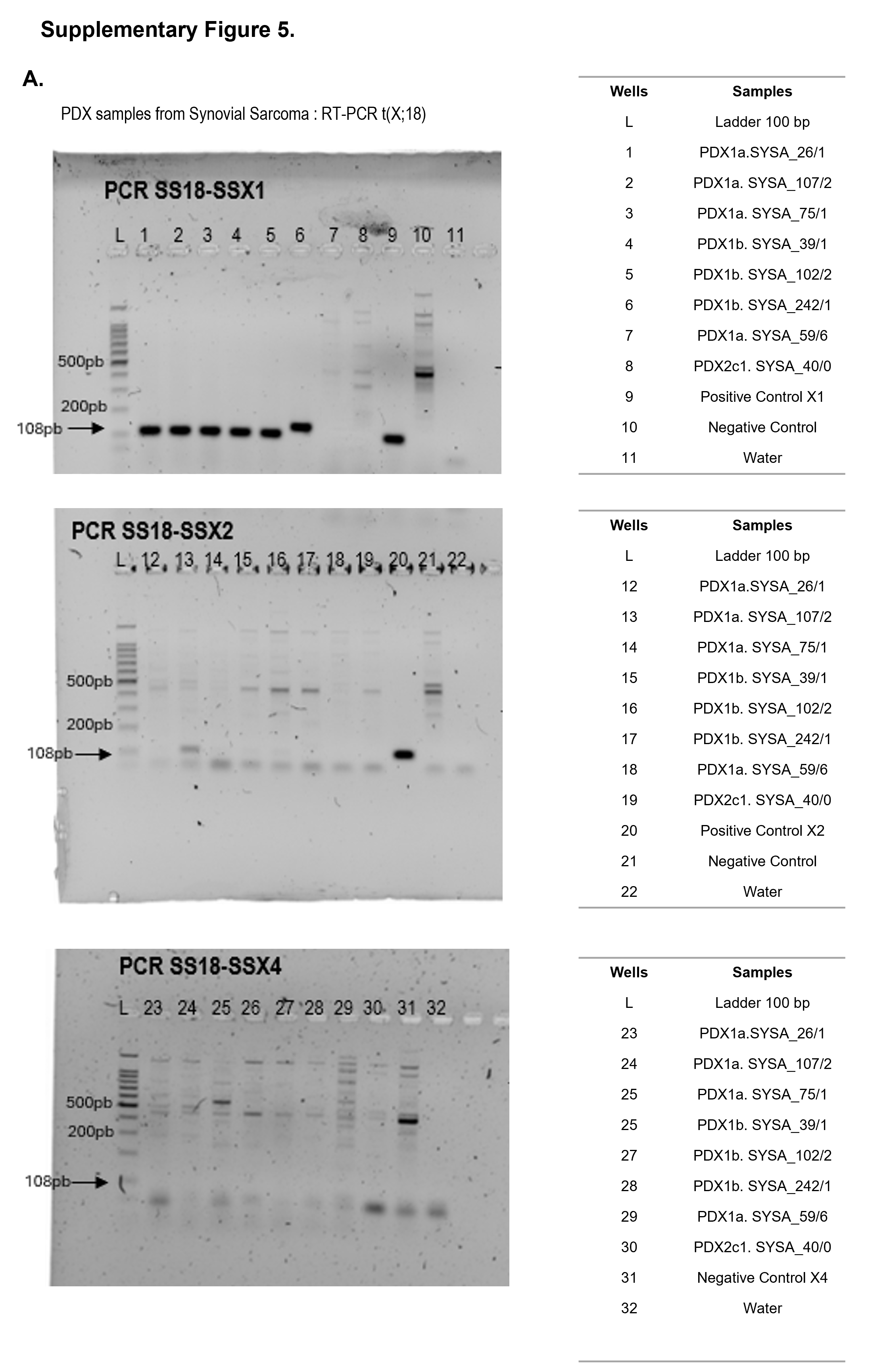

Supplement: Supplementary Figure 5.tif [file KCBT_A_2541974_SM8601.tif]

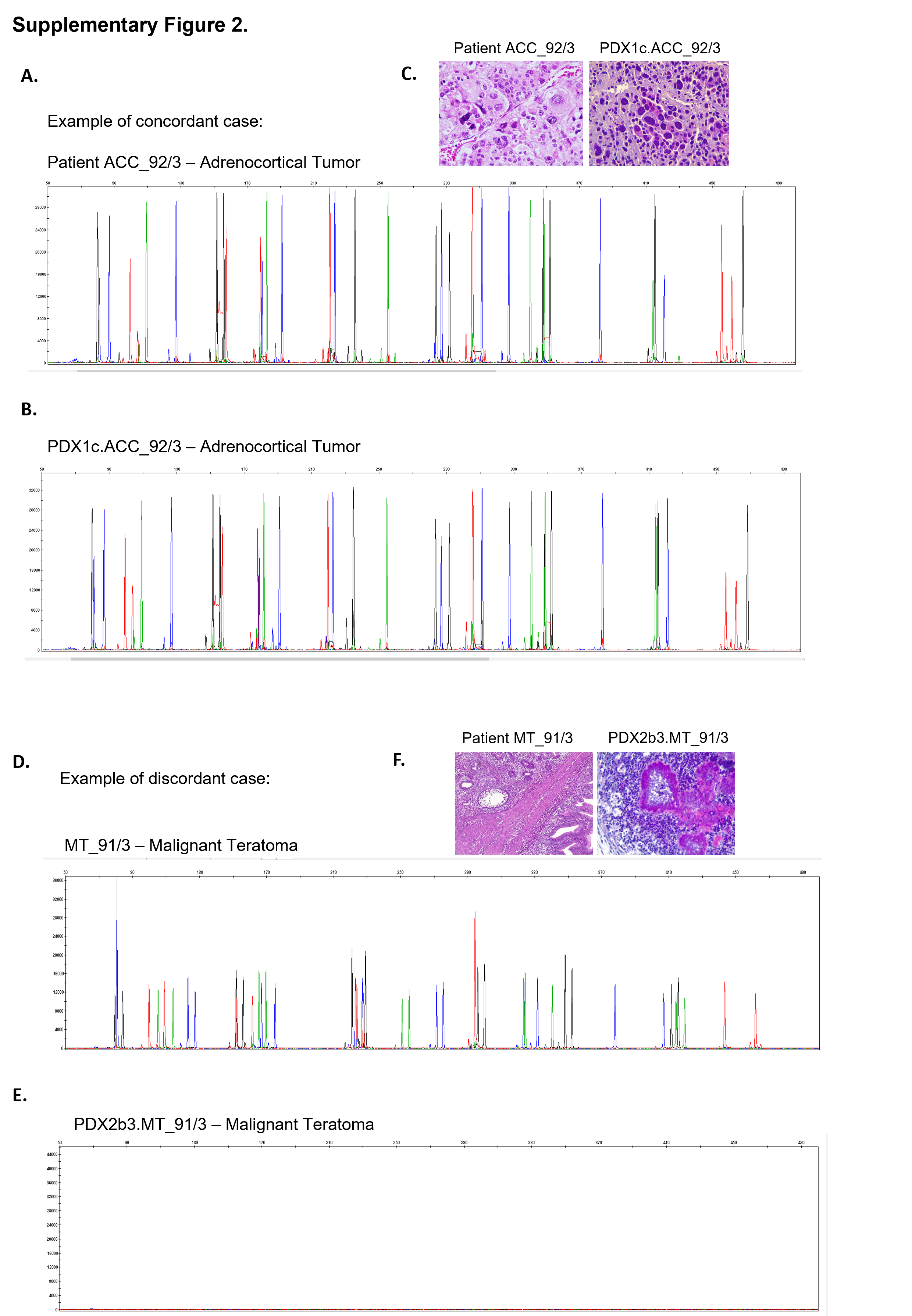

Supplement: Supplementary Figure 2.tif [file KCBT_A_2541974_SM8600.tif]

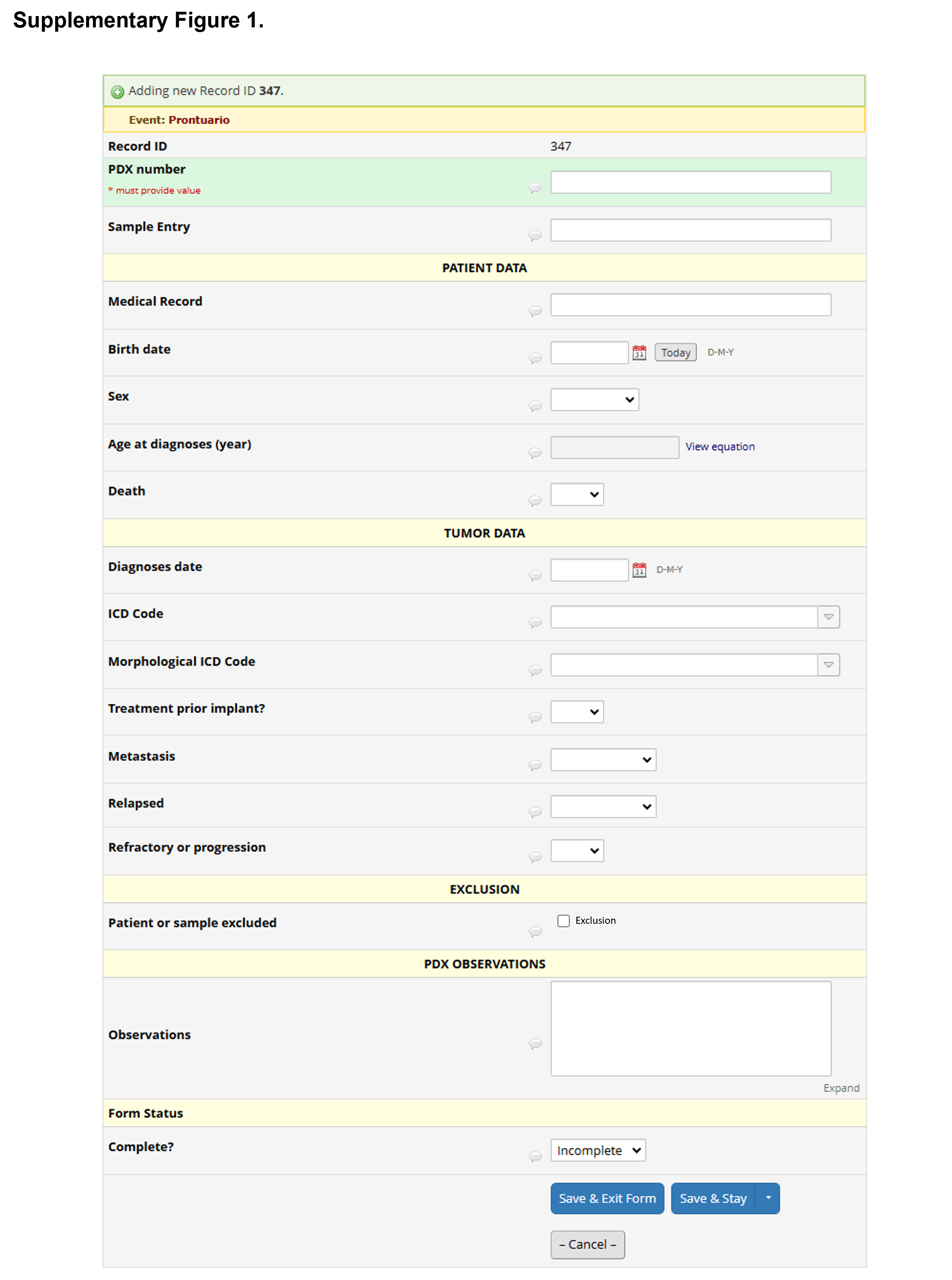

Supplement: Supplementary Figure 1.tif [file KCBT_A_2541974_SM8598.tif]

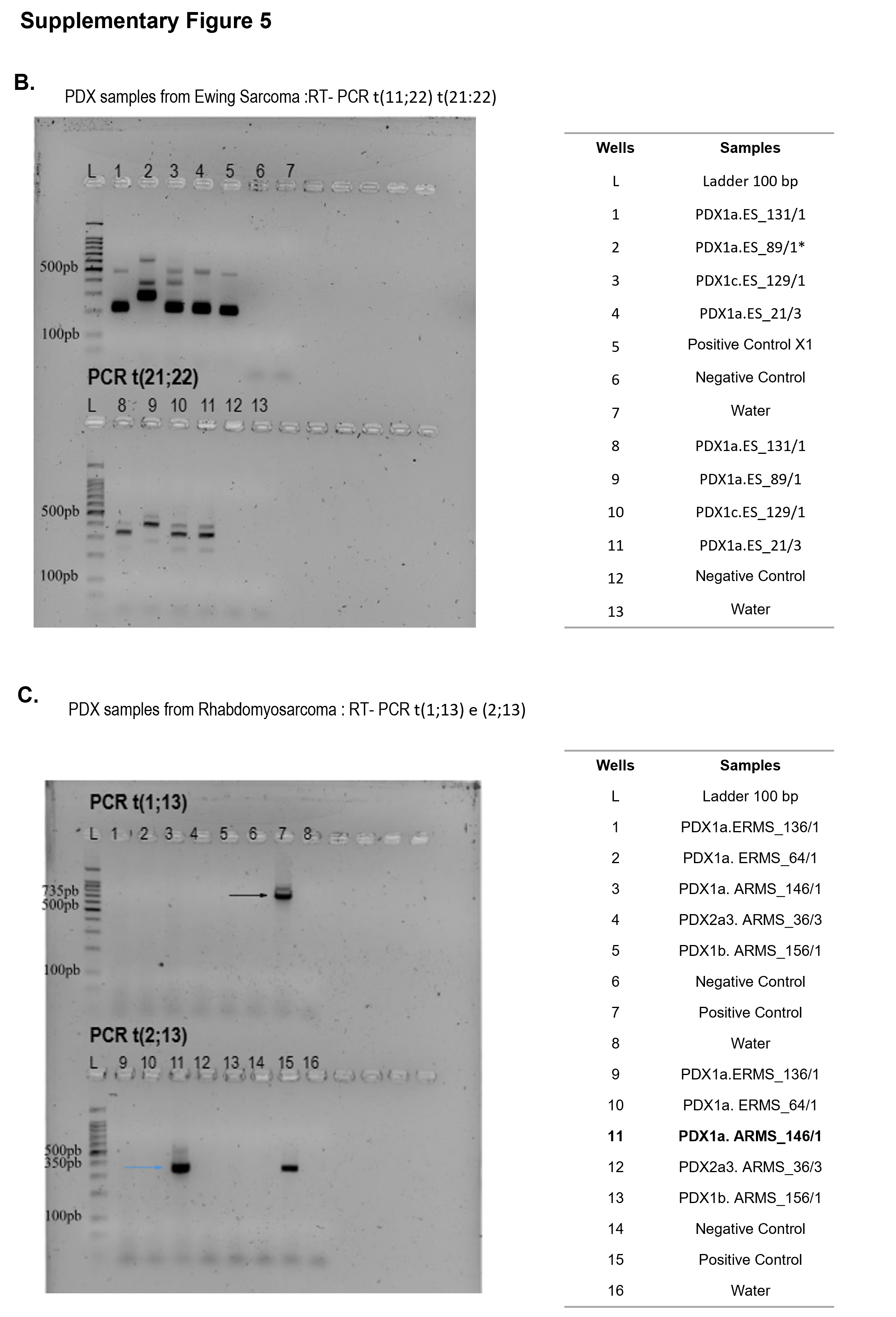

Supplement: Supplementary Figure 5_p2.tif [file KCBT_A_2541974_SM8596.tif]

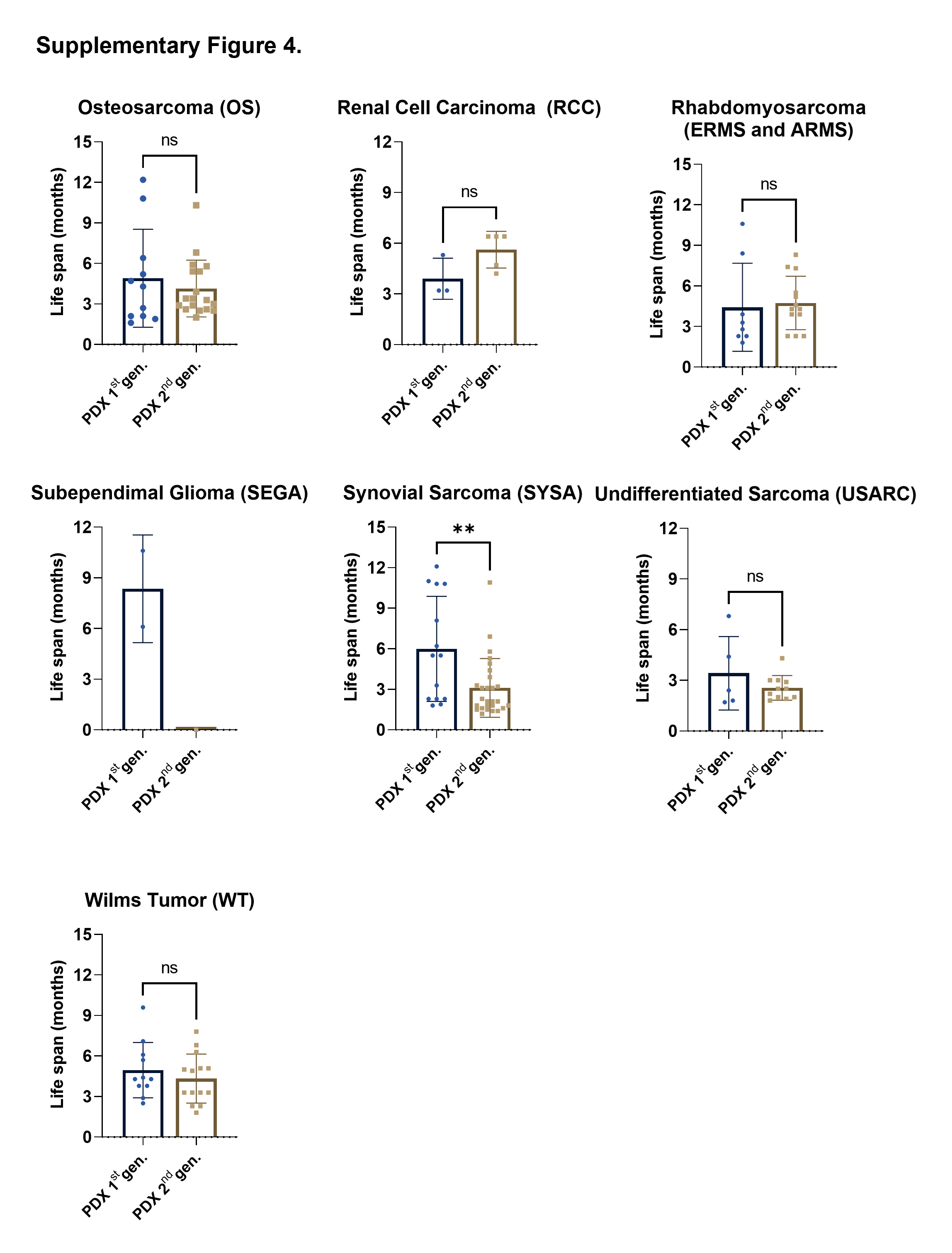

Supplement: Supplementary Figure 4_p2.tif [file KCBT_A_2541974_SM8595.tif]

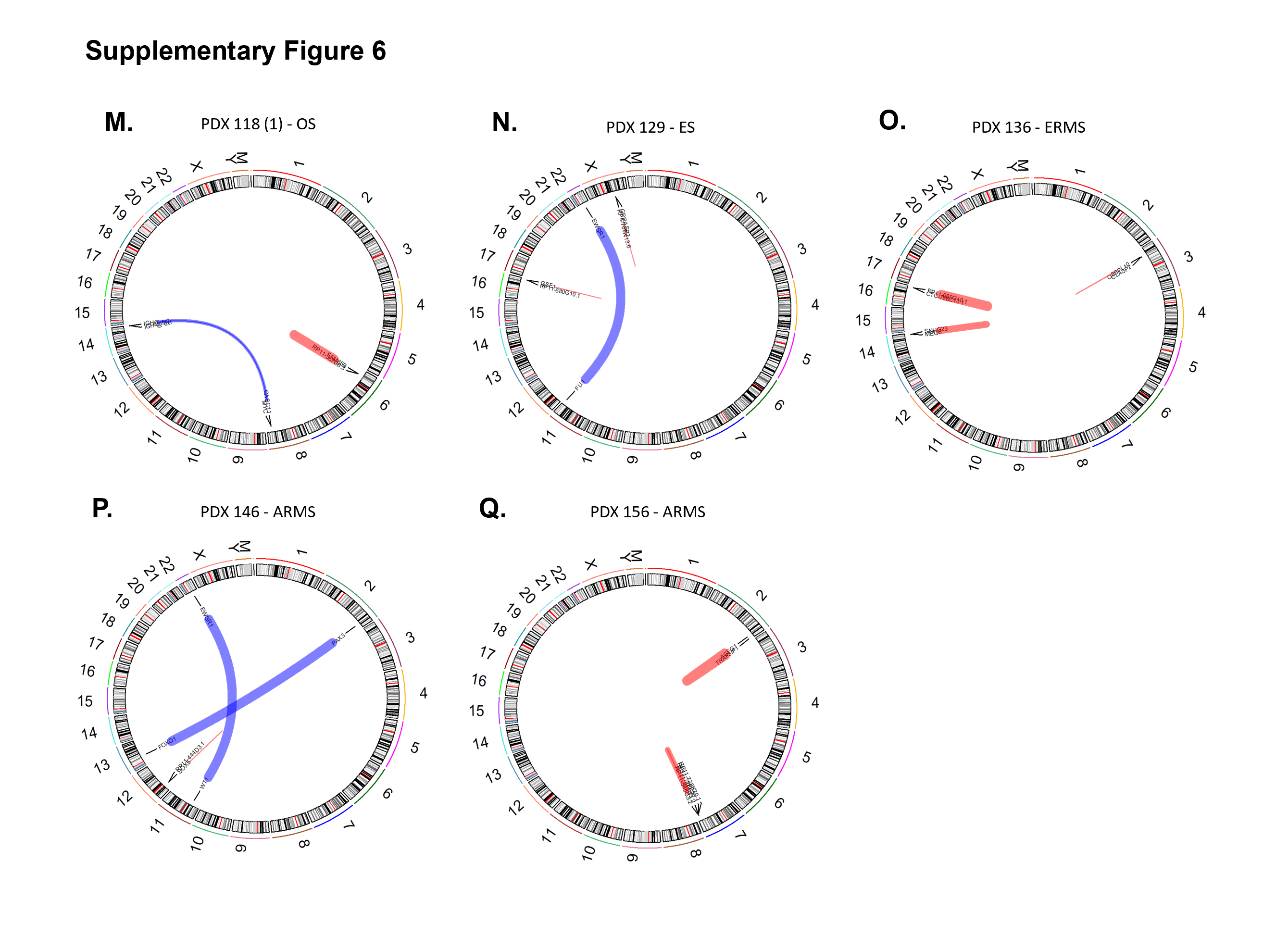

Supplement: Supplementary Figure 6_p2.tif [file KCBT_A_2541974_SM8594.tif]

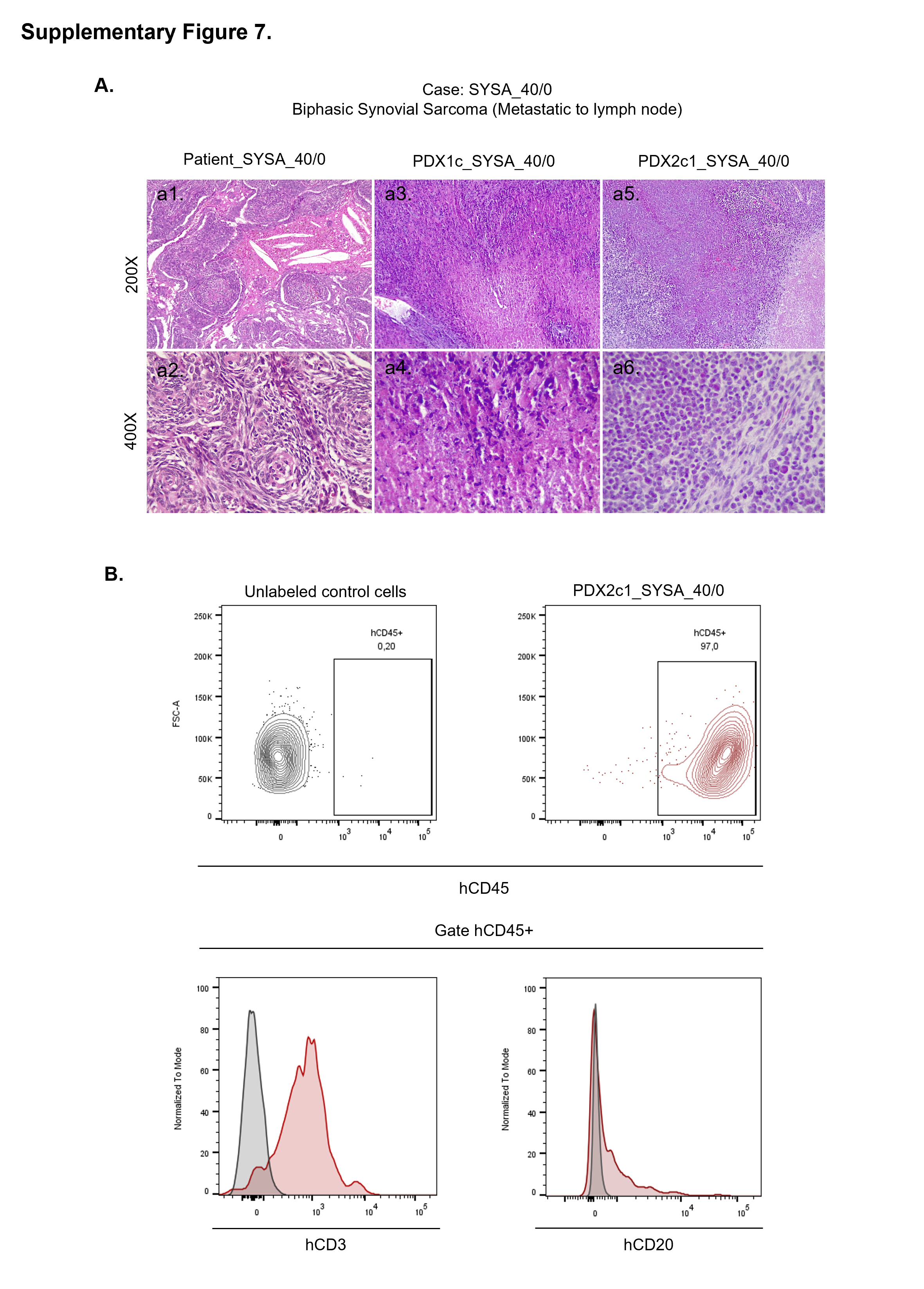

Supplement: Supplementary Figure 7.tif [file KCBT_A_2541974_SM8593.tif]

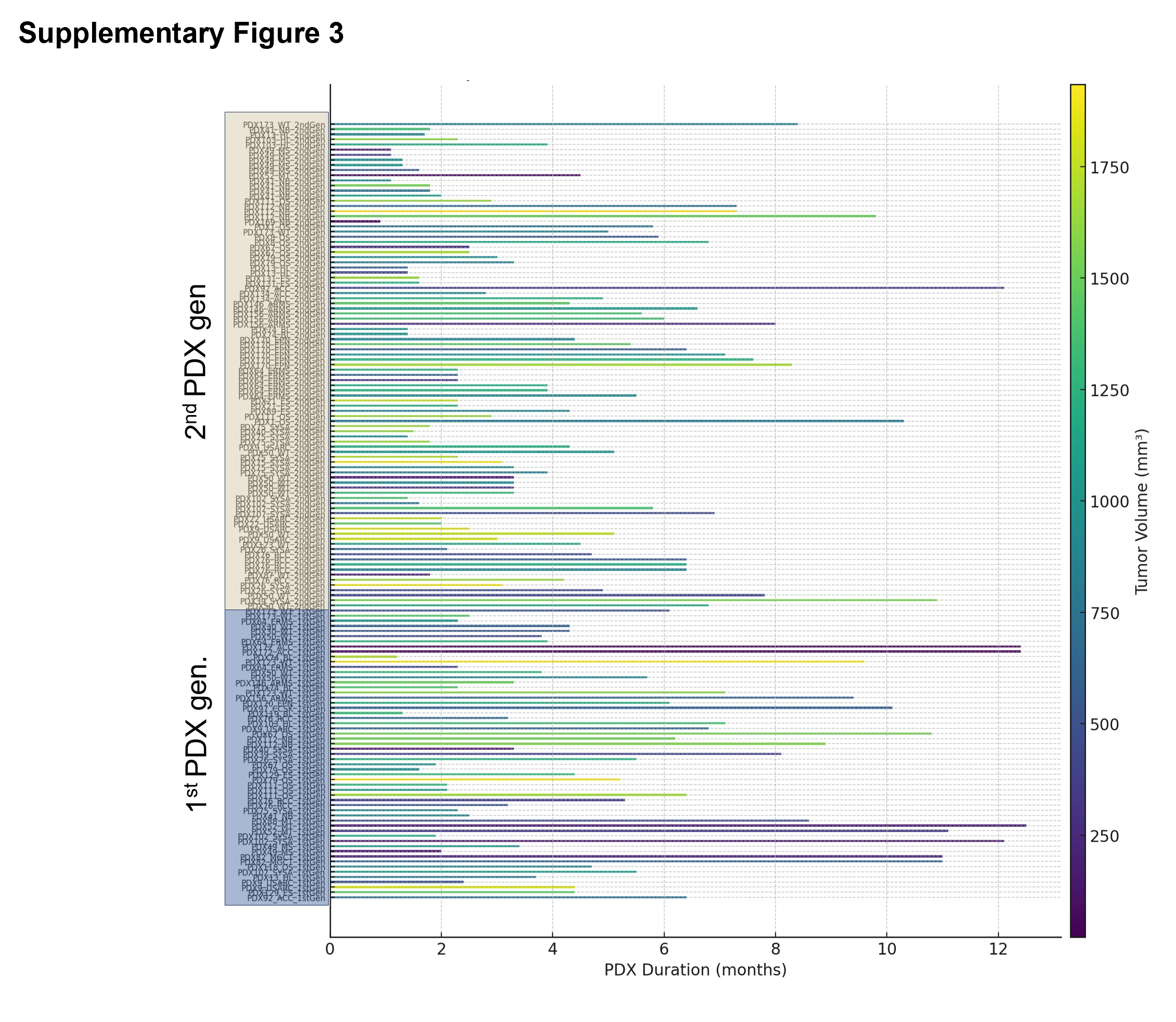

Supplement: Supplementary Figure 3.tif [file KCBT_A_2541974_SM8592.tif]

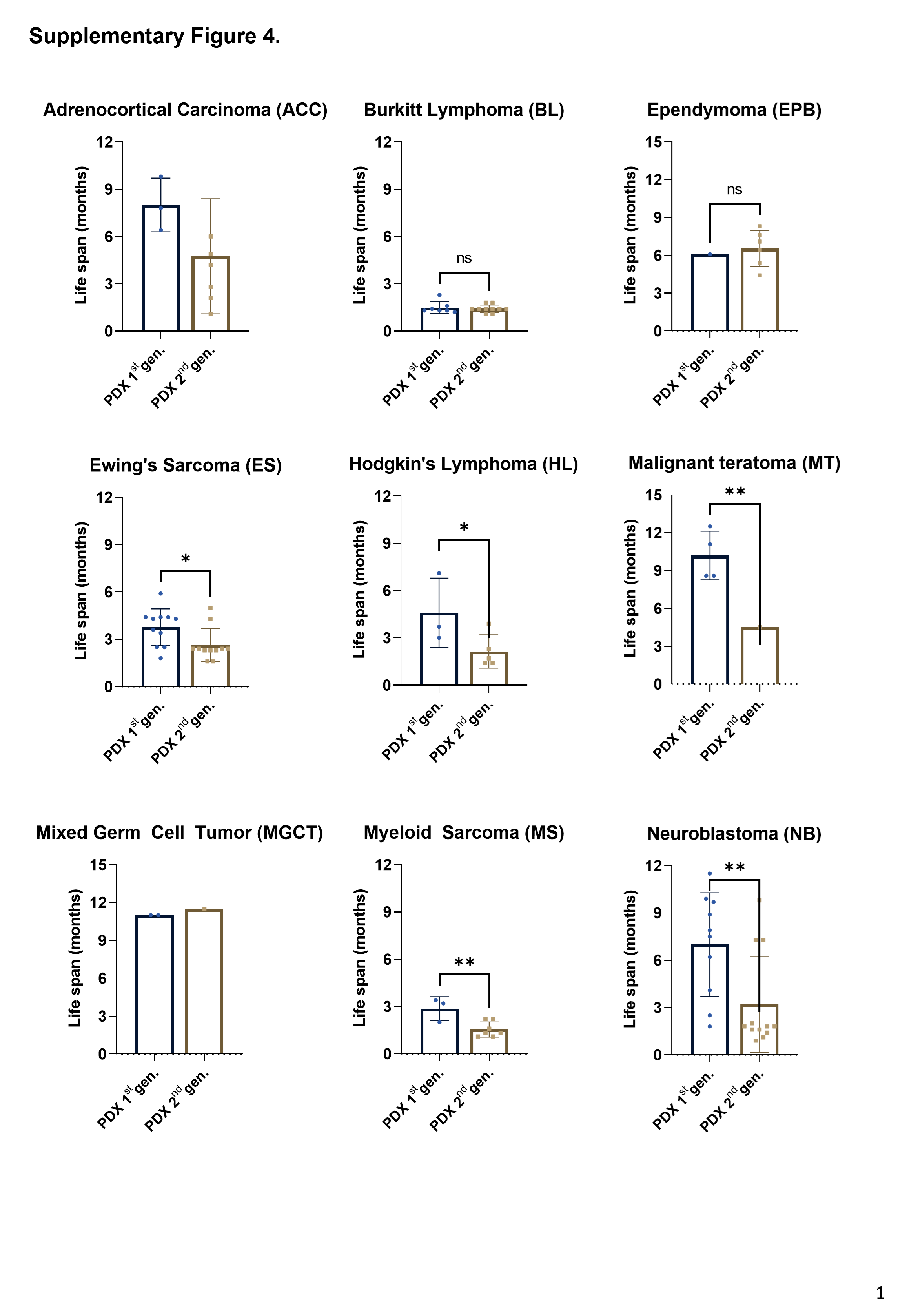

Supplement: Supplementary Figure 4.tif [file KCBT_A_2541974_SM8590.tif]
