# Supplementary material for: Establishing a pediatric solid tumor PDX biobank for precision oncology research
Source: Cancer Biol Ther. 2025 Aug 13;26(1):2541974. doi: 10.1080/15384047.2025.2541974 (PMC12351738; doi:10.1080/15384047.2025.2541974)
Supplement: Table S1.docx [file KCBT_A_2541974_SM8603.docx]

**Table S1. Primer sequences and PCR conditions.**

| Tumor types | Primers | |
| --- | --- | --- |
|  | **Name** | **Sequence** |
| Synovial Sarcoma | SSX1R (R) | 5´–ACACTCCCTTCGAATCATTTTCG– 3´ |
|  | SSX2R (R) | 5´–GCACTTCCTCCGAATCATTTC– 3´ |
|  | SSX4R (R) | 5´–GCACTTCCTTCAAACCATTTTCT– 3´ |
|  | SSX (F) | 5´–AGACCAACACAGCCTGGACCAC– 3´ |
| Alveolar Rhabdomyosarcoma | PAX7/FOXO1 (F) | 5´–TTTGAGAGGACCCACTACCC– 3´ |
|  | PAX3/FOXO1 (F) | 5´–AGACAGCTTTGTGCCTCCGTC– 3´ |
|  | FOXO1 (R) | 5´– CATCATTGCTGTGAGAGCCAG– 3´ |
| Ewing Sarcoma | EWSR1(F) | 5´–CCCACTAGTTACCCACCCCAAA– 3´ |
|  | EWSR1-FLI1(R) | 5´–AGGGTTGGCTAGGCGACTGCT– 3´ |
|  | EWSR1-ERG (R) | 5´–TGTTGGGTTTGCTCTTCCGCTC– 3´ |
|  | EWSR1-ERG3-FLI3 (F) | 5´–TCCTACAGCCAAGCTCCAAGTC– 3´ |
|  | EWSR1-FLI3 (R) | 5´–GTCGGGCCCAGGATCTGATAC– 3´ |
|  | EWSR1-ERG3 (F) | 5´–ACTCCCCGTTGGTGCCTTCC– 3´ |
|  |  |  |
|  |  |  |
| PCR conditions | | |
| Synovial sarcoma | | |
| 95ºC | 7 min | 1x |
| 94ºC | 45 sec | 10x (reduce 1ºC/cycle) |
| 66ºC | 45 sec |  |
| 72ºC | 1 min 30 sec |  |
| 94ºC | 45 sec | 30x |
| 56ºC | 45 sec |  |
| 72ºC | 1 min 30 sec |  |
| 72ºC | 5 min | 1x |
| 15ºC | holding | - |
| Alveolar rhabdomyosarcoma | | |
| Temperature | Time | Cycles |
| 94ºC | 2 min | 1x |
| 94ºC | 30 sec | 30x |
| 65ºC | 1 min |  |
| 72ºC | 2 min |  |
| 72ºC | 5min | 1x |
| 15ºC | holding | - |
| Ewing sarcoma - first and nested PCR | | |
| Temperature | Time | Cycles |
| 94ºC | 2 min | 1x |
| 94ºC | 30 sec | 20x for first and 30x for nested PCR |
| 68ºC | 1 min |  |
| 72ºC | 1 min |  |
| 72ºC | 2 min | 1x |
| 15ºC | holding | - |
