# Supplementary material for: Establishing a pediatric solid tumor PDX biobank for precision oncology research
Source: Cancer Biol Ther. 2025 Aug 13;26(1):2541974. doi: 10.1080/15384047.2025.2541974 (PMC12351738; doi:10.1080/15384047.2025.2541974)
Supplement: Table S3.docx [file KCBT_A_2541974_SM8599.docx]

**Table S3.** Descriptive data of tumor engraftment times across PDX models.
