# Supplementary material for: Establishing a pediatric solid tumor PDX biobank for precision oncology research
Source: Cancer Biol Ther. 2025 Aug 13;26(1):2541974. doi: 10.1080/15384047.2025.2541974 (PMC12351738; doi:10.1080/15384047.2025.2541974)
Supplement: Table S2.docx [file KCBT_A_2541974_SM8597.docx]

| **Origin** | **Sample** | **Histology** | **AMEL** | | **D1S1656** | | | | **D2S441** | | **D10S1248** | | **D13S317** | | **Penta E** | | **D16S539** | **D18S51 D2S1338** | | | | | **CSF1PO** | | **STR Markers**  **Penta D** | | **TH01** | | **vWA** | | | **D21S11 D7S820** | | | **D5S818** | | | **TPOX** | | **DYS391** | | **D8S1179** | | **D12S391** | | **D19S433** | | **FGA** | | **D22S1045** | | **PDX Status** | **Notes** |
| --- | --- | --- | --- | --- | --- | --- | --- | --- | --- | --- | --- | --- | --- | --- | --- | --- | --- | --- | --- | --- | --- | --- | --- | --- | --- | --- | --- | --- | --- | --- | --- | --- | --- | --- | --- | --- | --- | --- | --- | --- | --- | --- | --- | --- | --- | --- | --- | --- | --- | --- | --- | --- | --- |
| Human | P_SYSA_40/0 | SYSA | X | Y | 14 | 15 | 11 | 14 | 14 | 15 | 11 | 14 | 8 | 12 | 8 | 15 | 10 | 12 | 14 | 20 | 16 |  | 10 |  | 8 | 10 | 6 | 7 | | 15 | 16 | 28 | 30 | 10 | 12 | 11 | 12 | 8 | 10 | 10 |  | 10 | 12 | 18 | 18.3 | 12 | 14 | 19 | 24 | 16 | 17 |  |  |
| PDX | PDX2c1_SYSA_40/0 | SYSA | X | Y | 14 | 15 | 11 | 14 | 14 | 15 | 11 | 14 | 8 | 12 | 8 | 15 | 10 | 12 | 14 | 20 | 16 |  | 10 |  | 8 | 10 | 6 | 7 | | 15 | 16 | 28 | 30 | 10 | 12 | 11 | 12 | 8 | 10 | 10 |  | 10 | 12 | 18 | 18.3 | 12 | 14 | 19 | 24 | 16 | DO | Concordant | Drop Out (DO): |
| Human | P_ARMS_36/1 | ARMS | X |  | 18 | 19 | 11 | 15.3 | 10 | 12 | 14 | 15 | 8 | 11 | 13 | 17 | 11 | 12 | 14 | 15 | 24 | 26 | 10 | 11 | 12 | 13 | 7 | 9.3 | | 16 | 17 | 30.2 31.2 | | 11 |  | 11 | 12 | 9 |  |  |  | 12 | 13 | 19 | 21 | 13 |  | 20 | 21 | 11 | 16 |  |  |
| PDX | PDX2c1_ARMS_40/0 | ARMS | X |  | 18 | 19 | 11 | 15.3 | 10 | 12 | 14 | 15 | 8 | 11 | 13 | 17 | 11 | 12 | 14 | 15 | 24 | 26 | 10 | 11 | 12 | 13 | 7 | 9.3 | | 16 | 17 | 30.2 31.2 | | 11 |  | 11 | 12 | 9 |  |  |  | 12 | 13 | 19 | 21 | 13 |  | 20 | 21 | 16 | DO | Concordant | Drop Out (DO): |
| Human | P_EPN_170/0 | EPN | X | Y | 15 | 16 | 14 | 17.3 | 14 | 15 | 14 | 15 | 11 | 12 | 7 | 13 | 11 |  | 15 | 17 | 17 | 22 | 7 | 11 | 9 |  | 7 | 9.3 | | 16 | 17 | 28 | 30 | 9 | 11 | 12 | 13 | 8 | 9 | 11 |  | 12 | 14 | 19 |  | 14 | 16 | 22 | 23 | 15 | 16 |  |  |
| PDX | PDX2a2_EPN_170/4 | EPN | X | Y | 15 | 16 | 14 | 17.3 | 14 | 15 | 14 | 15 | 11 | 12 | 7 | 13 | 11 |  | 15 | 17 | 17 | 22 | 7 | 11 | 9 |  | 7 | 9.3 | | 16 | 17 | 28 | 30 | 9 | 11 | 12 | 13 | 8 | 9 | 11 |  | 12 | 14 | 19 |  | 14 | 16 | 22 | 23 | 15 | 16 | Concordant |  |
| Human | P_ES_129/1 | ES | X |  | 14 | 17 | 11 | 16 | 14 | 15 | 14 |  | 11 | 13 | 8 | 13 | 12 | 13 | 14 | 18 | 19 | 24 | 7 | 12 | 12 |  | 9 | 9.3 | | 14 | 17 | 30 33.2 | | 8 | 9 | 9 | 12 | 11 |  |  |  | 10 | 13 | 15 | 21 | 13 | 16.2 | 22 |  | 14 | 15 |  |  |
| PDX | PDX1a_ES_129/1 | ES | X |  | 14 | 17 | 11 | 16 | 14 | 15 | 14 |  | 11 | 13 | 8 | 13 | 12 | 13 | 14 | 18 | 19 | 24 | 7 | 12 | 12 |  | 9 | 9.3 | | 14 | 17 | 30 33.2 | | 8 | 9 | 9 | 12 | 11 |  |  |  | 10 | 13 | 15 | 21 | 13 | 16.2 | 22 |  | 14 | 15 | Concordant |  |
| Human | P_ES_21/0 | ES | X |  | 15 | 18 | 14 |  | 11 |  | 12 | 13 | 12 | 14 | 12 | 17 | 11 |  | 14 | 18 | 17 | 20 | 11 | 12 | 10 | 13 | 7 | 9 | | 16 | 17 | 28 | 29 | 8 | 10 | 11 | 12 | 8 | 11 |  |  | 12 | 14 | 21 | 22 | 13 | 13.2 | 23 | 25 | 11 | 15 |  |  |
| PDX | PDX2e1_ES_21/3 A | ES | X |  | 15 | 18 | 14 |  | 11 |  | 12 | 13 | 12 | 14 | 12 | 17 | 11 |  | 14 | 18 | 17 | 20 | 11 | 12 | 10 | 13 | 7 | 9 | | 16 | 17 | 28 | 29 | 8 | 10 | 11 | 12 | 8 | 11 |  |  | 12 | 14 | 21 | 22 | 13 | 13.2 | 23 | 25 | 11 | 15 | Concordant |  |
| Human | P_USARC_9/1 | USARC | X | Y | 14 | 15 | 16 | 17.1 | 11 | 14 | 14 | 15 | 11 | 12 | 12 | 13 | 10 | 11 | 17 | 18 | 18 | 20 | 10 | 13 | 9 | 12 | 8 | 9 | | 15 | 16 | 29 31.2 | | 8 |  | 11 | 13 | 8 | 11 | 10 |  | 13 | 14 | 17 | 21 | 13 | 14 | 23 | 24 | 17 |  |  |  |
| PDX | PDX2b3_USARC_9/1 | USARC | X | DO | 14 | 15 | 16 | 17.3 | 11 | 14 | 14 | 15 | 11 | 12 | 12 | DO | 10 | 11 | 17 | 18 | 18 | 20 | 10 | 13 | 9 | 12 | 8 | 9 | | 15 | 15.2 | 29 31.2 | | 8 |  | 11 | 13 | 8 | 11 | DO |  | 13 | 14 | 17 | 21 | 13 | 14 | 23 | 24 | 17 |  | Concordant | D1S1656 allele |
| Human | P_SYSA_39/1 | SYSA | X | Y | 17 | 18 | 12 | 16.3 | 11 |  | 12 | 14 | 8 | 11 | 7 | 11 | 9 | 10 | 14 |  | 20 | 21 | 10 | 13 | 11 | 12 | 8 |  | | 14 | 16 | 29 | 30 | 10 | 11 | 11 | 12 | 10 |  | 11 |  | 13 | 14 | 15 | 19.3 | 13 | 15 | 20 | 25 | 15 | 17 |  |  |
| PDX | PDX2b3_SYSA_39/1 | SYSA | X | Y | 17 | 18 | 12 | 16.3 | 11 | 12 | 12 | 14 | 8 | 11 | 7 | 11 | 7 | 9 | 9 11.2 | | 20 | 21 | 10 | 13 | 11 | 12 | 8 |  | | 14 | 16 | 29 | 30 | 5 | 9.2 | 11 | 12 | 10 |  | 11 |  | 13 | 14 | 15 | 19.3 | 13 | 14 | 20 | 21 | 15 | 17 | Concordant | D2S441 allele 12; |
| PDX | PDX2b2_SYSA_39/2 | SYSA | X | Y | 17 | 18 | 12 | 16.3 | 11 |  | 12 | 14 | 8 | 11 | 7 | 11 | 9 | 10 | 14 |  | 20 | 21 | 10 | 13 | 11 | 12 | 8 |  | | 14 | 16 | 29 | 30 | 10 | 11 | 11 | 12 | 10 |  | 11 |  | 13 | 14 | 14 | 19.3 | 13 | 15 | 20 | 25 | 15 | 17 | Concordant | D12S391 allele 14 |
| Human | P_SYSA_43/20 | SYSA | X | Y | 14 | 15 | 13 |  | 12 | 13 | 14 | 15 | 11 | 12 | 8 | 14 | 9 | 13 | 13 | 18 | 17 | 23 | 10 | 11 | 2.2 | 8 | 7 |  | | 15 | 19 | 29 32.2 | | 10 |  | 11 | 12 | 8 |  | 10 |  | 10 | 14 | 17.3 | 22 | 13.2 | 14 | 23 | 24 | 15 | 16 |  |  |
| PDX | PDX2a1_SYSA_43/6 | SYSA | X | Y | 14 | 15 | 13 |  | 12 | 13 | 14 | 15 | 11 | 12 | 8 | 14 | 9 | DO | 13 | 19 | 17 | 23 | 10 | 11 | 2.2 | 8 | 7 |  | | 15 | 19 | 29 32.2 | | 10 |  | 11 | 12 | 8 |  | 10 |  | 10 | 14 | 17.3 | 22 | 13.2 | 14 | 23 | 24 | 15 | 16 | Concordant | Drop Out (DO): |
| Human | P_OS_5/20 | OS | X | Y | 15 | 16 | 14 | 15 | 11 | 14 | 14 | 15 | 11 |  | 7 | 12 | 11 | 12 | 12 | 18 | 18 | 25 | 11 | 12 | 11 | 13 | 6 | 7 | | 14 | 15 | 28 | 31 | 9 | 13 | 11 | 12 | 8 | 11 | 10 |  | 12 | 13 | 22 |  | 11 | 14 | 21 | 22 | 15 |  |  |  |
| PDX | PDX2a_OS_5/0 | OS |  |  |  |  |  |  |  |  |  |  |  |  |  |  |  |  |  |  |  |  |  |  |  |  |  |  | |  |  |  |  |  |  |  |  |  |  |  |  |  |  |  |  |  |  |  |  |  |  | Discordant | No peaks |
| Human | P_OS_1/1 | OS | X |  | 15 | 16 | 15 | 15.3 | 11 | 14 | 14 | 16 | 12 | 15 | 7 | 8 | 12 | 13 | 16 | 17 | 16 | 20 | 11 | 12 | 3.2 | 13 | 6 |  | | 15 | 17 | 30 32.2 | | 8 | 11 | 12 | 13 | 8 |  |  |  | 14 | 15 | 19 | 20 | 13 | 15.2 | 21 | 23 | 11 | 16 |  |  |
| PDX | PDX2a1_OS_1/1 | OS | X |  | 15 | DO | 15 | 15.3 | 11 | 14 | 16 | DO | 12 | DO | 7 | 8 | 12 | 13 | 16 | 17 | 16 | DO | 11 | 12 | 3.2 | 13 | 6 |  | | 15 | 17 | 30 32.2 | | 8 | 11 | 13 | DO | 8 |  |  |  | 14 | DO | 19 | 20 | 13 | 15.2 | 21 | 23 | 11 | DO | Concordant | discrepancies: |
| PDX | PDX2b1_OS_1/4 | OS | X |  | 15 | DO | 15.3 | DO | 11 | 14 | 14 | 16 | 12 | DO | 7 | 8 | 12 | 13 | 16 | 17 | 16 | DO | 11 | DO | 3.2 | 13 | 6 |  | | 15 | 17 | 30 32.2 | | 8 | 11 | 13 | DO | 8 |  |  |  | 14 | 15 | 19 | 20 | 13 | 15.2 | 21 | 23 | 11 | DO | Concordant | discrepancies: |
| Human | P_ARMS_146/1 | ARMS |  | Y |  | 16 | 11 | 16 | 10 | 14 | 13 | 16 | 12 | 13 | 7 | 13 | 12 | 13 | 10 | 13 | 20 | 23 | 10 | 13 | 11 | 12 | 9 | 9.3 | | 16 | 19 | 27 | 29 | 11 |  | 11 | 13 | 8 | 11 | 10 |  | 12 | 14 | 15 | 21 | 12 | 13 | 19 | 21 | 15 | 16 |  |  |
| PDX | PDX1c_ARMS_146/1 | ARMS | X | DO | 14 | 17 | 11 | 16 | 14 | DO | 14 | DO | 11 | 13 | 8 | 13 | 12 | 13 | 14 | 18 | 19 | 24 | 7 | 12 | 12 | DO | 9 | 9.3 | | 14 | 17 | 30 33.2 | | 8 | 9 | 9 | 12 | 11 | DO | DO |  | 10 | 13 | 15 | 21 | 13 | 16.2 | 22 | DO | 14 | 15 | Concordant | markers: |
| Human | P_SEGA_4/1 | SEGA | X | Y | 17 | 18 | 15.3 | 17.3 | 14 |  | 13 | 16 | 11 | 13 | 10 | 16 | 11 | 13 | 15 | 18 | 17 | 18 | 10 | 11 | 10 | 12 | 6 | 8 | | 16 | 17 | 28.2 30.2 | | 7 | 11 | 10 | 13 | 11 | 12 | 10 |  | 13 |  | 22 |  | 12.2 | 16 | 23 | 26 | 15 |  |  |  |
| PDX | PDX1c_SEGA_4/1 | SEGA |  |  |  |  |  |  |  |  |  |  |  |  |  |  |  |  |  |  |  |  |  |  |  |  |  |  | |  |  |  |  |  |  |  |  |  |  |  |  |  |  |  |  |  |  |  |  |  |  | Discordant | No peaks |
| Human | P_ES_14/1 | ES | X |  | 16 | 17 | 12 |  | 10 | 11 | 13 |  | 10 | 11 | 11 | 12 | 12 | 13 | 12 | 17 | 18 | 24 | 11 | 12 | 10 | 11 | 6 | 7 | | 16 | 18 | 28 33.2 | | 9 |  | 12 |  | 11 | 12 |  |  | 10 | 14 | 15 | 18 | 15 | 15.2 | 23 | 24 | 11 | 15 |  |  |
| PDX | PDX2a3_ES_14/1 | ES | X |  | 16 | 17 | 12 |  | 10 | 11 | 13 |  | 10 | 11 | 11 | 12 | 12 | 13 | 12 | 17 | 18 | 24 | 11 | 12 | 10 | 11 | 6 | 7 | | 16 | 18 | 28 33.2 | | 9 |  | 12 |  | 11 | 12 |  |  | 10 | 14 | 15 | 18 | 15 | 15.2 | 23 | 24 | 11 | 15 | Concordant |  |
| Human | P_HL_13/2 | HL | X | Y | 17 | 18 | 12 | 16 | 10 | 11.3 | 14 | 16 | 8 | 12 | 8 | 14 | 11 |  | 16 | 17 | 20 | 25 | 11 | 12 | 9 | 12 | 6 |  | | 15 | 16 | 29 | 30 | 8 | 11 | 8 | 10 | 8 | 11 | 11 |  | 11 | 14 | 19 | 23 | 14.2 | 15 | 23 |  | 10 | 11 |  |  |
| PDX | PDX2b4_HL_13/2 | HL | X | Y | 17 | 18 | 12 | 16 | 10 | 11.3 | 14 | 16 | 8 | 12 | 8 | 14 | 11 |  | 16 | 17 | 20 | 25 | 11 | 12 | 9 | 12 | 6 |  | | 15 | 16 | 29 | 30 | 8 | 11 | 8 | 10 | 8 | 11 | 11 |  | 11 | 14 | 19 | 23 | 14.2 | 15 | 23 | 24 | 10 | 11 | Concordant | FGA allele 24 |
| Human | P_USARC_26/1 | USARC | X |  | 16 | 18 | 15 | 16 | 11 | 12 | 15 | 16 | 8 | 9 | 8 | 12 | 9 | 13 | 14 | 18 | 17 |  | 11 | 12 | 10 | 12 | 7 | 8 | | 16 | 18 | 29 |  | 11 |  | 11 | 13 | 9 | 11 |  |  | 13 | 14 | 20 | 22 | 14 |  | 20 | 23 | 11 | 14 |  |  |

**Table S2.** Raw data from short-tandem repeat analysis.

.

| PDX | PDX2a1_USARC_26/1 | USARC | X |  | 16 | 18 | 15 | 16 | 11 | 12 | 15 | 16 | 8 | 9 | 8 | 12 | 9 | 13 | 14 | 18 | 17 |  | 11 | 12 | 10 | 12 | 7 | 8 | 16 | 18 | 28 | 29 | 11 |  | 11 | 13 | 9 | 11 |  | 13 | 14 | 20 | 22 | 14 |  | 20 | 23 | 11 | 14 | Concordant | D21S11 allele 28 |
| --- | --- | --- | --- | --- | --- | --- | --- | --- | --- | --- | --- | --- | --- | --- | --- | --- | --- | --- | --- | --- | --- | --- | --- | --- | --- | --- | --- | --- | --- | --- | --- | --- | --- | --- | --- | --- | --- | --- | --- | --- | --- | --- | --- | --- | --- | --- | --- | --- | --- | --- | --- |
| Human | P_NB_41/1 | NB | X |  | 16 | 19 | 12 | 14 | 12 | 14 | 13 | 14 | 11 | 12 | 7 | 17 | 10 | 13 | 14 | 20 | 17 | 25 | 8 | 12 | 11 | 12 | 7 | 9.3 | 15 | 16 | 28 | 29 | 8 | 10 | 11 | 14 | 8 |  |  | 13 | 14 | 15 | 21 | 13 | 15 | 20 | 23 | 14 | 15 |  |  |
| PDX | PDX2a3.NB_41/3 | NB | X |  | 16 | 19 | 12 | 14 | 12 | 14 | 13 | 14 | 11 | 12 | 7 | 17 | 10 | 13 | 14 | 20 | 17 | 25 | 12 | DO | 11 | 12 | 7 | 9.3 | 15 | 16 | 28 | 29 | 8 | 10 | 14 | DO | 8 |  |  | 13 | 14 | 15 | 21 | 13 | 15 | 20 | 23 | 14 | 15 | Concordant | discrepancies: |
| Human | P_MT_88/1 | MT | X | Y | 15 | 17 | 10 | 16 | 10 | 15 | 14 | 15 | 9 | 12 | 12 | 16 | 12 |  | 14 | 17 | 21 | 23 | 10 | 15 | 9 | 11 | 6 | 9 | 16 | 17 | 30 | 32.2 | 10 | 12 | 11 | 12 | 8 | 9 | 10 | 8 | 16 | 15 | 19 | 13 | 14 | 21 | 22 | 17 |  |  |  |
| PDX | PDX1b_MT_88/4 | MT | X | Y | 15 | 17 | 10 | 16 | 10 | 15 | 14 | 15 | 9 | 12 | 12 | 16 | 12 |  | 14 | 17 | 21 | 23 | 10 | 15 | 9 | 11 | 6 | 9 | 17 | DO | 30 | 32.2 | 10 | 12 | 11 | 12 | 8 | 9 | 10 | 8 | 16 | 15 | 19 | 13 | 14 | 21 | 22 | 17 |  | Concordant | Drop Out (DO): |
| Human | P_HB_23/0 | HB | X |  | 15 | 18 | 13 | 18.3 | 11.3 | 14 | 13 | 15 | 11 |  | 7 | 11 | 9 | 12 | 12 | 14 | 19 | 20 | 10 | 13 | 13 |  | 9.3 |  | 15 | 19 | 31.2 | 32.2 | 10 |  | 11 | 12 | 8 |  |  | 14 |  | 17 | 19 | 15 |  | 20 | 23 | 11 | 16 |  |  |
| PDX | PDX1c_HB_23/0 | HB |  |  |  |  |  |  |  |  |  |  |  |  |  |  |  |  |  |  |  |  |  |  |  |  |  |  |  |  |  |  |  |  |  |  |  |  |  |  |  |  |  |  |  |  |  |  |  | Discordant | No peaks |
| Human | P_SYSA_107/2 | SYSA | X |  | 15 | 17 | 10 | 16 | 12 | 14 | 14 | 15 | 12 |  | 15 | 16 | 11 | 13 | 12 | 20 | 25 |  | 11 | 12 | 12 |  | 7 | 8 | 17 |  | 29 | 30 | 8 |  | 11 | 13 | 8 | 11 |  | 14 |  | 17 |  | 13 |  | 22 |  | 11 | 17 |  |  |
| PDX | PDX2a2_SYSA_107/2 | SYSA | X |  | 15 | 17 | 10 | 16 | 12 | 14 | 14 | 15 | 12 |  | 15 | 16 | 11 | DO | 12 | 20 | 25 |  | 11 | 12 | 12 |  | 7 | DO | 17 |  | 29 | 30 | 8 |  | 11 | 13 | 8 | 11 |  | 14 |  | 17 |  | 13 |  | 22 |  | 11 | 17 | Concordant | discrepancies: |
| Human | P_MS_49/1 | MS | X | Y | 16 | 17 | 15 | 16.3 | 11 |  | 14 |  | 9 | 11 | 12 | 15 | 9 | 12 | 12 | 13 | 24 |  | 11 | 12 | 10 | 12 | 6 | 9.3 | 16 | 19 | 28 | 32.2 | 8 | 9 | 9 | 11 | 8 |  | 12 | 10 | 14 | 15 | 18 | 13 |  | 23 | 28 | 16 | 17 |  |  |
| PDX | PDX2b2_MS_49/1 | MS | X | DO | 17 | DO | 15 | 16.3 | 11 |  | 14 |  | 11 | DO | 15 | DO | 9 | 12 | 12 | 13 | 24 |  | 11 | 12 | 10 | 12 | 6 | 9.3 | 19 | DO | 28 | 32.2 | 8 | 9 | 9 | 11 | 8 |  | DO | 10 | 14 | 18 | DO | 13 |  | 28 | DO | 16 | 17 | Concordant | discrepancies: |
| Human | P_SEGA_27/1 | SEGA | X |  | 17 |  | 12 | 16 | 11 |  | 13 | 14 | 9 | 11 | 8 | 13 | 11 | 12 | 11 | 17 | 24 | 25 | 10 |  | 13 | 14 | 7 | 9.3 | 15 | 17 | 30 |  | 11 | 12 | 9 | 10 | 8 | 11 |  | 14 | 15 | 17 | 18 | 12 | 13.2 | 23 | 24 | 14 | 16 |  |  |
| PDX | PDX1a._SEGA_27/1 | SEGA | X |  | 17 |  | 12 | 16 | 11 |  | 13 | 14 | 9 | 11 | 8 | 13 | 11 | 12 | 11 | 17 | 24 | 25 | 10 |  | 13 | 14 | 7 | 9.3 | 15 | 17 | 30 |  | 11 | 12 | 9 | 10 | 8 | 11 |  | 14 | 15 | 17 | 18 | 12 | 13.2 | 23 | 24 | 14 | 16 | Concordant |  |
| Human | P_OS_29/1 | OS | X |  | 15 | 16 | 12 | 16 | 13 | 14 | 14 | 16 | 8 |  | 11 |  | 9 | 11 | 12 | 16 | 24 | 25 | 10 | 11 | 9 | 12 | 9.3 |  | 16 | 16.1 | 29 | 30 | 10 | 12 | 11 | 12 | 9 | 11 |  | 11 | 12 | 20 | 23 | 14 | 16 | 22 | 24 | 15 | 17 |  |  |
| PDX | PDX2a1_OS_29/1 | OS |  |  |  |  |  |  |  |  |  |  |  |  |  |  |  |  |  |  |  |  |  |  |  |  |  |  |  |  |  |  |  |  |  |  |  |  |  |  |  |  |  |  |  |  |  |  |  | Discordant | No peaks |
| Human | P_WT_51/2 | WT | X | Y | 15 | 17 | 12 | 16.3 | 11 | 12 | 15 | 16 | 11 | 13 | 14 | 18 | 10 | 11 | 14 | 16 | 19 | 21 | 10 | 11 | 9 | 14 | 9.3 |  | 16 | 19 | 30 | 31.2 | 8 | 10 | 11 |  | 11 |  | 10 | 12 |  | 19.3 | 20 | 14 | 15.2 | 21 | 24 | 15 |  |  |  |
| PDX | PDX2a1_WT_51/2 | WT |  |  |  |  |  |  |  |  |  |  |  |  |  |  |  |  |  |  |  |  |  |  |  |  |  |  |  |  |  |  |  |  |  |  |  |  |  |  |  |  |  |  |  |  |  |  |  | Discordant | No peaks |
| Human | P_NB_81/1 | NB | X | Y | 15 |  | 13 | 17.3 | 10 | 14 | 15 |  | 11 | 12 | 11 | 21 | 9 | 12 | 13 | 14 | 17 | 24 | 10 | 11 | 8 | 11 | 7 | 9 | 15 | 16 | 28 | 35 | 11 | 12 | 9 | 11 | 8 | 9 | 10 | 13 | 14 | 17 | 20 | 14 | 14.2 | 20 | 25 | 15 | 16 |  |  |
| PDX | PDX1c._NB_81/1 | NB |  |  |  |  |  |  |  |  |  |  |  |  |  |  |  |  |  |  |  |  |  |  |  |  |  |  |  |  |  |  |  |  |  |  |  |  |  |  |  |  |  |  |  |  |  |  |  | Discordant | No peaks |
| Human | P_HB_72/1 | HB | X | Y | 16 | 17 | 10 | 16 | 11.3 | 14 | 13 | 14 | 11 | 13 | 11 | 15 | 9 | 11 | 12 | 18 | 19 | 20 | 12 | 13 | 9 |  | 6 | 9.3 | 15 | 18 | 29 | 31.2 | 9 | 12 | 12 | 13 | 6 | 8 | 11 | 14 | 16 | 18 | 19.3 | 13.2 | 14 | 25 | 26 | 14 | 15 |  |  |
| PDX | PDX1c._HB_72/1 | HB |  |  |  |  |  |  |  |  |  |  |  |  |  |  |  |  |  |  |  |  |  |  |  |  |  |  |  |  |  |  |  |  |  |  |  |  |  |  |  |  |  |  |  |  |  |  |  | Discordant | No peaks |
| Human | P_WT_50/2 | WT | X |  | 17 | 18 | 12 | 16 | 14 |  | 14 |  | 12 |  | 14 | 15 | 12 | 13 | 13 | 14 | 18 | 23 | 7 | 11 | 11 | 13 | 7 | 8 | 15 | 18 | 28 | 29 | 9 | 11 | 10 | 12 | 8 | 11 |  | 12 | 13 | 20 | 24 | 13 | 14 | 18 | 20 | 16 |  |  |  |
| PDX | PDX2a1_WT_50/2 | WT | X |  | 17 | 18 | 12 | 16 | 14 |  | 14 |  | 12 |  | 14 | 15 | 12 | 14 | 13 | 14 | 18 | 23 | 7 | 11 | 11 | 13 | 7 | 8 | 15 | 18 | 28 | 29 | 9 | 11 | 10 | 12 | 8 | 11 |  | 12 | 13 | 20 | 24 | 13 | 14 | 18 | 20 | 16 |  | Concordant | D16S539 allele 14 |
| Human | P_MT_52/1 | MT | X |  | 16 | 17 | 16.3 | 18 | 11 | 14 | 13 | 14 | 11 | 12 | 11 | 13 | 8 | 9 | 13 | 14 | 20 | 22 | 10 |  | 10 | 13 | 6 |  | 17 | 18 | 29 | 30 | 11 | 12 | 11 | 12 | 11 | 12 |  | 11 | 14 | 18 | 20 | 14 | 15.2 | 21 | 24 | 15 | 17 |  |  |
| PDX | PDX1c_MT_52/1 | MT | X |  | 16 | 17 | 16.3 | 18 | 11 | 14 | 13 | 14 | 11 | 12 | 11 | 13 | 8 | 9 | 13 | 14 | 20 | 22 | 10 |  | 10 | 13 | 6 |  | 17 | 18 | 29 | 30 | 11 | 12 | 11 | 12 | 11 | 12 |  | 11 | 14 | 18 | 20 | 14 | 15.2 | 21 | 24 | 15 | 17 | Concordant |  |
| Human | P_BL_119/12 | BL | X |  | 14 | 17 | 13 | 17.3 | 11 | 14 | 14 |  | 11 | 12 | 8 | 12 | 11 | 12 | 12 | 15 | 17 | 19 | 8 | 11 | 8 |  | 6 | 7 | 15 | 18 | 29 | 32.2 | 11 | 12 | 9 | 13 | 8 | 10 |  | 14 | 15 | 15 | 18 | 13.2 | 14 | 23 | 24 | 11 | 16 |  |  |
| PDX | PDX2a1_BL_119/12 | BL | X |  | 14 | DO | 13 | DO | 11 | 14 | 14 |  | 11 | 12 | 8 | 12 | 11 | 12 | 12 | 15 | 17 | 19 | 8 | 11 | 8 |  | 6 | 7 | 15 | 18 | 29 | 32.2 | 11 | 12 | 9 | 13 | 8 | 10 |  | 14 | 15 | 15 | 18 | 13.2 | 14 | 23 | 24 | 11 | 16 | Concordant | discrepancies: |
| Human | P_NB_112/1 | NB | X |  | 14 | 16 | 14 | 17 | 11 | 15 | 14 | 15 | 9 | 12 | 11 | 18 | 12 |  | 14 | 15 | 17 | 19 | 12 | 14 | 11 | 12 | 6 | 9.3 | 16 | 17 | 30 | 31 | 8 | 10 | 11 |  | 10 | 11 |  | 13 | 14 | 17 | 20 | 14 |  | 23 | 24 | 15 |  |  |  |
| PDX | PDX2c1_NB_112/1 | NB | X |  | 14 | 16 | 14 | 17 | 11 | 15 | 14 | 15 | 9 | 12 | 11 | 18 | 12 |  | 14 | 15 | 17 | DO | 12 | 14 | 11 | 12 | 6 | 9.3 | 16 | 17 | 30 | 31 | 8 | 10 | 11 |  | 10 | 11 |  | 13 | 14 | 17 | 20 | 14 |  | 23 | 24 | 15 |  | Concordant | Drop Out (DO): |
| Human | P_RCC_76/1 | RCC | X |  | 16 | 18 | 14 | 15 | 12 | 14 | 14 | 15 | 11 | 12 | 11 | 15 | 11 | 13 | 17 | 18 | 22 | 24 | 10 |  | 14 | 15 | 6 | 8 | 17 | 18 | 28 | 30 | 7 | 10 | 11 | 13 | 8 | 11 |  | 14 | 15 | 19 | 20 | 15 | 16 | 23 | 25 | 11 | 16 |  |  |
| PDX | PDX2c2_RCC_76/1 | RCC | X |  | 16 | 18 | 14 | 15 | 12 | 14 | 14 | 15 | 11 | 12 | 11 | 15 | 13 | DO | 17 | 18 | 22 | 24 | 10 |  | 14 | 15 | 6 | 8 | 17 | 18 | 28 | 30 | 7 | 10 | 11 | 13 | 8 | 11 |  | 14 | 15 | 19 | 20 | 15 | 16 | 23 | 25 | 11 | 16 | Concordant | Drop Out (DO): |
| Human | P_ERMS_64/1 | ERMS | X | Y | 15 | 18 | 14 | 15 | 10 | 12 | 13 |  | 8 | 9 | 13 | 14 | 10 | 11 | 12 | 15 | 17 | 23 | 11 | 12 | 10 | 12 | 9.3 |  | 16 | 16.1 | 30 | 33.2 | 6.3 | 10 | 11 | 12 | 9 | 11 | 10 | 14 | 15 | 22 | 23 | 13 | 14 | 20 | 25 | 15 |  |  |  |
| PDX | PDX2a1_ERMS_64/1 | ERMS | X | Y | 15 | 18 | 14 | 15 | 10 | 12 | 13 |  | 8 | 9 | 13 | 14 | 10 | 11 | 12 | 15 | 17 | 23 | 11 | 12 | 10 | 12 | 9.3 |  | 16 | 16.1 | 30 | 33.2 | 6.3 | 10 | 11 | 12 | 9 | 11 | 10 | 14 | 15 | 22 | 23 | 13 | 14 | 20 | 25 | 15 |  | Concordant |  |

| Human | P_OS_67/1 | OS | X |  | 14 |  | 12 | 17.3 | 10 | 11 | 11 | 15 | 11 | 13 | 10 | 11 | 11 |  | 15 | 16 | 16 | 26 | 12 |  | 9 | 12 | 6 | 9.3 | 17 | 18 | 29 | 30 | 10 | 11 | 7 | 13 | 8 | 11 |  | 11 | 15 | 17 | 20 | 12 |  | 20 | 22 | 16 | 17 |  | |
| --- | --- | --- | --- | --- | --- | --- | --- | --- | --- | --- | --- | --- | --- | --- | --- | --- | --- | --- | --- | --- | --- | --- | --- | --- | --- | --- | --- | --- | --- | --- | --- | --- | --- | --- | --- | --- | --- | --- | --- | --- | --- | --- | --- | --- | --- | --- | --- | --- | --- | --- | --- |
| PDX | PDX2c2_OS_67/1 | OS | X |  | 14 |  | 17.3 | DO | 10 | 11 | 11 | 15 | 11 | 13 | 11 | DO | 11 |  | 15 | 16 | 16 | 26 | 12 |  | 9 | 12 | 6 | 9.3 | 18 | DO | 29 | 30 | 10 | 11 | 7 | DO | 8 | 11 |  | 11 | 15 | 17 | DO | 12 |  | 20 | 22 | 16 | 17 | Concordant | Drop Out (DO): |
| Human | P_SYSA_75/1 | SYSA | X |  | 13 | 14 | 13 | 17.3 | 11 | 14 | 13 | 15 | 9 | 12 | 11 | 12 | 11 | 12 | 13 |  | 19 | 21 | 10 | 13 | 5 | 12 | 7 | 9 | 15 | 17 | 27 | 28 | 9 | 11 | 11 | 12 | 6 | 8 |  | 12 |  | 15 | 17 | 14 | 15 | 22 | 24 | 15 | 17 |  |  |
| PDX | PDX2b2_SYSA_75/1 | SYSA | X |  | 13 | 14 | 13 | 17.3 | 11 | 14 | 13 | 15 | 9 | 12 | 11 | 12 | 11 | 12 | 13 |  | 19 | 21 | 10 | 13 | 5 | 12 | 7 | 9 | 15 | 17 | 27 | 28 | 9 | 11 | 11 | 12 | 6 | 8 |  | 12 |  | 15 | 17 | 14 | 15 | 22 | 24 | 15 | 17 | Concordant |  |
| Human | P_WT_87/0 | WT | X | Y | 14 | 17 | 13 | 17.3 | 10 | 11 | 13 | 14 | 11 | 13 | 7 | 12 | 11 | 14 | 12 | 15.3 | 21 | 23 | 10 | 13 | 8 | 13 | 9 |  | 17 | 20 | 28 | 30 | 10 | 11 | 12 |  | 8 | 11 | 11 | 10 | 14 | 19 | 19.3 | 13.2 | 14 | 21 | 22 | 16 |  |  |  |
| PDX | PDX2c2_WT_87/0 | WT | X | Y | 14 | 17 | 13 | 17.3 | 10 | 11 | 13 | 14 | 13 | DO | 7 | 12 | 11 | 14 | 12 | 16 | 21 | 23 | 10 | 13 | 8 | 13 | 9 |  | 17 | 20 | 28 | 30 | 10 | 11 | 12 |  | 8 | 11 | 11 | 10 | 14 | 19 | 19.3 | 13.2 | 14 | 21 | 22 | 16 |  | Concordant | discrepancies: |
| Human | P_ES_89/1 | ES | X |  | 16 | 18 | 13 | 17 | 10 | 13 | 13 | 15 | 11 | 12 | 12 | 17 | 12 | 13 | 15 | 16 | 18 | 19 | 10 | 12 | 11 | 13 | 6 | 9 | 16 | 17 | 29 | 31 | 8 | 9 | 13 | 14 | 9 | 12 |  | 11 | 14 | 18 | 19 | 13.2 | 15.2 | 22 | 23 | 15 | 16 |  |  |
| PDX | PDX2a1.P_ES_89/1 | ES | X |  | 16 | 18 | 13 | 17 | 10 | 13 | 13 | 15 | 11 | 12 | 12 | 17 | 12 | 13 | 15 | 16 | 18 | 19 | 10 | 12 | 11 | 13 | 6 | 9 | 16 | 17 | 29 | 31 | 8 | 9 | 13 | 14 | 9 | 12 |  | 11 | 14 | 18 | 19 | 13.2 | 15.2 | 22 | 23 | 15 | 16 | Concordant |  |
| Human | P_WT_82/1 | WT | X |  | 15 | 17 | 17 |  | 10 | 11 | 13 | 14 | 9 | 13 | 15 | 16 | 11 |  | 17 | 18 | 22 | 23 | 12 |  | 9 | 11 | 7 | 9.3 | 16 | 19 | 28 | 30 | 10 | 11 | 12 |  | 8 | 11 |  | 11 | 15 | 19 | 20 | 13 |  | 23 | 26 | 17 |  |  |  |
| PDX | PDX1b_WT_82/1 | WT | X |  | 15 | 17 | 17 |  | 10 | 11 | 13 | 14 | 9 | 13 | 15 | 16 | 11 |  | 17 | 18 | 22 | 23 | 12 |  | 9 | 11 | 7 | 9.3 | 16 | 19 | 28 | 30 | 10 | 11 | 12 |  | 8 | 11 |  | 11 | 15 | 19 | 20 | 13 |  | 23 | 26 | 17 |  | Concordant |  |
| Human | P_WT_86/3 | WT | X |  | 15 | 16 | 11 | 13 | 11 | 12 | 13 | 14 | 11 | 13 | 7 | 13 | 11 | 13 | 15 | 16 | 17 | 18 | 7 | 11 | 9 | 10 | 6 | 7 | 16 | 17 | 28 | 30 | 11 | 12 | 12 | 13 | 8 | 9 |  | 13 | 15 | 17.3 | 20 | 13 | 14 | 21 |  | 11 | 17 |  |  |
| PDX | PDX2b3_WT_86/3 | WT |  |  |  |  |  |  |  |  |  |  |  |  |  |  |  |  |  |  |  |  |  |  |  |  |  |  |  |  |  |  |  |  |  |  |  |  |  |  |  |  |  |  |  |  |  |  |  | Discordant | No peaks |
| Human | P_CCSK_97/1 | CCSK | X |  | 16 |  | 13 | 15 | 10 | 12 | 12 | 13 | 10 | 14 | 5 | 14 | 12 |  | 17 | 18 | 23 |  | 11 | 12 | 2.2 | 10 | 6 |  | 14 | 16 | 31.2 | 32.2 | 10 | 11 | 11 | 12 | 8 | 9 |  | 12 | 14 | 18 | 23 | 13 | 14 | 21 | 24 | 15 | 16 |  |  |
| PDX | PDX1c_CCSK_97/1 | CCSK | X |  | 16 |  | 13 | 15 | 10 | 12 | 12 | 13 | 10 | 14 | 5 | 14 | 12 |  | 17 | 18 | 23 |  | 11 | 12 | 2.2 | 10 | 6 |  | 14 | 16 | 31.2 | 32.2 | 10 | 11 | 11 | 12 | 8 | 9 |  | 12 | 14 | 18 | 23 | 13 | 14 | 21 | 24 | 15 | 16 | Concordant |  |
| Human | P_HD_103/0 | HD | X |  | 15 | 16 | 16 |  | 14 | 15 | 15 | 16 | 9 | 12 | 8 | 12 | 8 | 12 | 15 | 16 | 18 | 23 | 11 |  | 11 | 13 | 6 |  | 18 |  | 29 |  | 8 | 9 | 11 |  | 8 |  |  | 12 | 14 | 19 | 21 | 13 | 15 | 21 | 24 | 15 | 16 |  |  |
| PDX | PDX2c1_HD_103/0 | HD | X |  | 15 | 16 | 16 |  | 14 | 15 | 15 | 16 | 9 | 12 | 8 | 12 | 8 | 12 | 15 | 16 | 18 | 23 | 11 |  | 11 | 13 | 6 |  | 18 |  | 29 |  | 8 | 9 | 11 |  | 8 |  |  | 12 | 14 | 19 | 21 | 13 | 15 | 21 | 24 | 15 | 16 | Concordant |  |
| Human | P_WT_123/1 | WT | X |  | 17 | 18 | 14 | 15.3 | 11 | 14 | 13 |  | 9 |  | 7 | 15 | 11 | 12 | 14 |  | 17 | 19 | 11 | 12 | 9 | 14 | 9 |  | 16 | 17 | 30 | 30.2 | 8 | 9 | 10 | 11 | 10 | 11 |  | 12 | 13 | 18 | 22 | 13 | 14.2 | 19 | 20 | 11 | 17 |  |  |
| PDX | PDX2a2_WT_123/1 | WT | X |  | 17 | 18 | 14 | 15.3 | 11 | 14 | 13 |  | 9 |  | 7 | 15 | 11 | 12 | 14 |  | 17 | 19 | 11 | 12 | 9 | 14 | 9 |  | 16 | 17 | 30 | 30.2 | 8 | 9 | 10 | 11 | 10 | 11 |  | 12 | 13 | 18 | 22 | 13 | 14.2 | 19 | DO | 17 | DO | Concordant | discrepancies: |
| Human | P_ERMS_136/1 | ERMS | X |  | 15 | 16 | 12 | 16 | 11 | 11.3 | 13 | 15 | 8 | 13 | 11 | 12 | 11 | 12 | 18 | 24 | 23 | 24 | 12 | 14 | 12 | 13 | 7 | 9.3 | 17 | 18 | 28 | 31 | 9 | 12 | 11 | 12 | 8 | 9 |  | 7 | 14 | 20 | 21 | 13 | 14 | 23 | 24 | 15 |  |  |  |
| PDX | PDX1a_ERMS_136/1 | ERMS | X |  | 15 | 16 | 12 | 16 | 11 | 11.3 | 13 | 15 | 8 | 13 | 11 | 12 | 11 | 12 | 18 | 24 | 23 | 24 | 12 | 14 | 12 | 13 | 7 | DO | 17 | 18 | 28 | 31 | 9 | 12 | 11 | 12 | 8 | 9 |  | 7 | 14 | 20 | 21 | 13 | 14 | 23 | 24 | 15 |  | Concordant | Drop Out (DO): |
| Human | P_ARMS_156/1 | ARMS | X |  | 18 |  | 14 | 17.3 | 14 |  | 14 |  | 11 | 12 | 9 | 13 | 13 |  | 12 | 14 | 24 | 26 | 12 |  | 11 | 14 | 7 | 9.3 | 14 | 17 | 29 | 33.2 | 11 | 12 | 12 | 13 | 8 |  |  | 12 | 14 | 17 | 22 | 14 |  | 18 | 22 | 11 | 16 |  |  |
| PDX | PDX1c._ARMS_156/1 | ARMS | X |  | 18 |  | 14 | 17.3 | 14 |  | 14 |  | 11 | 12 | 9 | 13 | 13 |  | 12 | 14 | 24 | DO | 12 |  | 11 | 14 | 7 | DO | 14 | 17 | 29 | 33.2 | 11 | 12 | 12 | 13 | 8 |  |  | 12 | 14 | 17 | 22 | 14 |  | 18 | 22 | 11 | 16 | Concordant | discrepancies: |
| Human | P_NB_169/1 | NB | X | Y | 14 | 16 | 13 | 17.3 | 10 | 14 | 14 |  | 11 | 12 | 11 | 12 | 9 | 11 | 15 |  | 19 |  | 10 | 12 | 9 | 13 | 9 | 9.3 | 17 | 19 | 29 | 31 | 9 | 10 | 10 | 12 | 9 | 10 | 11 | 11 | 12 | 18 | 20 | 14 | 15 | 21 | 23 | 15 | 16 |  |  |
| PDX | PDX1c_NB_169/1 | NB | X | Y | 14 | 16 | 13 | 17.3 | 10 | 14 | 14 |  | 11 | 12 | 11 | 12 | 9 | 11 | 15 |  | 19 |  | 10 | 12 | 9 | 13 | 9 | DO | 17 | 19 | 29 | 31 | 9 | 10 | 10 | 12 | 9 | 10 | 11 | 11 | 12 | 18 | 20 | 14 | 15 | 21 | 23 | 15 | 16 | Concordant | Drop Out (DO): |
| Human | P_WT_173/1 | WT | X | Y | 17 |  | 12 | 15 | 11 |  | 15 | 17 | 9 | 13 | 5 | 12 | 9 |  | 16 | 17 | 22.2 | 24 | 9 | 13 | 8 |  | 6 | 7 | 16 |  | 27 | 28 | 8 | 10 | 10 | 11 | 8 |  | 9 | 12 |  | 17 | 23 | 13 | 14 | 19 | 25 | 16 |  |  |  |
| PDX | PDX2b3_WT_173/1 | WT | X | Y | 17 |  | 12 | 15 | 11 |  | 15 | 17 | 9 | DO | 5 | 12 | 9 |  | 16 | 17 | 22.2 | 24 | 9 | 13 | 8 |  | 6 | 7 | 16 |  | 27 | 28 | 8 | 10 | 10 | 11 | 8 |  | 9 | 12 |  | 17 | 23 | 13 | 14 | 19 | 25 | 16 |  | Concordant | Drop Out (DO): |
| Human | P_OS_8/1 | OS | X |  | 14 |  | 16 |  | 11 | 14 | 15 |  | 8 | 11 | 10 | 11 | 11 | 12 | 14 | 17 | 20 |  | 10 | 12 | 12 | 17 | 7 | 9.3 | 16 | 18 | 29 | 30.2 | 9 |  | 10 | 13 | 8 | 11 |  | 13 | 15 | 15 |  | 13 |  | 23 |  | 15 |  |  |  |
| PDX | PDX1a_OS_8/1 | OS | X |  | 14 |  | 16 |  | 11 | 14 | 15 |  | 8 | DO | 10 | 11 | 12 | DO | 13.3 | 17 | 20 |  | 10 | 12 | 12 | 17 | 7 | DO | 16 | DO | 29 | 30.2 | 9 |  | 10 | 13 | 8 | 11 |  | 13 | 15 | 15 |  | 13 |  | 23 |  | 15 |  | Concordant | D18S51 allele |
| Human | P_ERMS_14/1 | ERMS | X | Y | 15 | 17 | 11 | 14 | 11 |  | 14 |  | 8 |  | 7 |  | 9 |  | 15 | 17 | 25 |  | 12 |  | 2.2 | 12 | 7 |  | 16 | 19 | 30 | 31 | 8 | 9 | 10 | 11 | 12 |  |  | 12 | 14 | 18 | 24 | 13 | 14 | 22 |  | 10 | 15 |  |  |
| PDX | PDX2b3_ERMS_14/1 | ERMS |  |  |  |  |  |  |  |  |  |  |  |  |  |  |  |  |  |  |  |  |  |  |  |  |  |  |  |  |  |  |  |  |  |  |  |  |  |  |  |  |  |  |  |  |  |  |  | Discordant | No peaks |
| Human | P_USARC_22/1 | USARC | X |  | 17 | 18 | 14 | 15 | 11 | 12 | 13 | 14 | 12 | 13 | 11 | 12 | 9 | 11 | 14 | 16 | 17 |  | 10 | 11 | 2.2 | 10 | 7 |  | 15 | 16 | 30 | 31 | 8 | 10 | 7 | 11 | 7 | 8 |  | 12 | 13 | 18 | 19 | 12.2 | 14 | 20 | 21 | 11 | 15 |  |  |
| PDX | PDX2b3_USARC_22/1 | USARC | X |  | 17 | 18 | 14 | 15 | 11 | 12 | 13 | 14 | 12 | 13 | 11 | 12 | 9 | 11 | 14 | 16 | 17 |  | 10 | 11 | 2.2 | 10 | 7 |  | 15 | 16 | 30 | 31 | 8 | 10 | 7 | 11 | 7 | 8 |  | 12 | 13 | 18 | DO | 12.2 | 14 | 20 | 21 | 11 | 15 | Concordant | Drop Out (DO): |
| Human | P_WT_30/1 | WT |  |  |  |  |  |  |  |  |  |  |  |  |  |  |  |  |  |  |  |  |  |  |  |  |  |  |  |  |  |  |  |  |  |  |  |  |  |  |  |  |  |  |  |  |  |  |  |  | Without reference |

| PDX | PDX2c1_WT_30/1 | WT | X | Y | 15 |  | 15 |  | 10 | 11 | 13 | 15 | 8 | 12 | 11 | 15 | 9 | 11 | 12 | 17 | 17 | 22 | 11 | 12 | 9 |  | 7 | 9.3 | 16.1 | 17 | 30 | 31 | 10 |  | 11 |  | 11 | 12 | 10 | 15 |  | 17.3 | 19 | 13 | 15 | 20 | 23 | 15 | 16 | Concordant |  |
| --- | --- | --- | --- | --- | --- | --- | --- | --- | --- | --- | --- | --- | --- | --- | --- | --- | --- | --- | --- | --- | --- | --- | --- | --- | --- | --- | --- | --- | --- | --- | --- | --- | --- | --- | --- | --- | --- | --- | --- | --- | --- | --- | --- | --- | --- | --- | --- | --- | --- | --- | --- |
| Human | P_BL_74/0 | BL | X | Y | 15 | 17 | 12 | 14 | 11 | 14 | 13 | 14 | 10 | 13 | 11 | 12 | 9 | 12 | 15 | 16 | 19 | 23 | 10 | 11 | 9 | 12 | 9.3 |  | 17 | 18 | 28 | 30 | 10 |  | 11 | 12 | 8 |  | 10 | 13 | 14 | 17 | 19 | 12.2 | 14 | 20 | 25 | 15 |  |  |  |
| PDX | PDX2c1_BL_74/0 | BL | X | Y | 15 | 17 | 12 | 14 | 11 | 14 | 13 | 14 | 10 | 13 | 11 | 12 | 9 | 12 | 15 | 16 | 19 | 23 | 10 | 11 | 9 | 12 | 9.3 |  | 17 | 18 | 28 | 30 | 10 |  | 11 | 12 | 8 |  | 10 | 13 | 14 | 17 | 19 | 12.2 | 14 | 20 | 25 | 15 |  | Concordant |  |
| Human | P_OS_79/3 | OS | X | Y | 14 | 18 | 14 | 15 | 11 | 14 | 14 |  | 11 |  | 14 | 16 | 9 | 13 | 14 | 18 | 20 | 24 | 11 |  | 8.2 | 10 | 7 | 8 | 15 | 17 | 27 | 28 | 9.1 | 12 | 11 | 12 | 8 |  |  | 13 | 14 | 16 | 22 | 12 | 15 | 19.2 | 21 | 15 |  |  |  |
| PDX | PDX2b2_OS_79/3 | OS | X | DO | DO | 18 | 14 | 15 | 11 | DO | 14 |  | 11 |  | 14 | 16 | DO | 13 | DO | 18 | 20 | 24 | 11 |  | 8.2 | 10 | DO | 8 | 15 | 17 | DO | 28 | 9 | 12 | 11 | 12 | 8 |  |  | 13 | 14 | 16 | 22 | 12 | 15 | 19.2 | 21 | 15 |  | Concordant | discrepancies: |
| Human | P_ACC_92/1 | ACC | X | Y | 17 | 18 | 13 | 15 | 11 | 14 | 13 | 16 | 8 | 11 | 12 | 13 | 12 | 13 | 12 | 16 | 20 |  | 12 |  | 8 | 13 | 8 | 9.3 | 17 | 18 | 28 | 30 | 10 |  | 10 | 11 | 8 |  |  | 14 |  | 17 | 22 | 13 | 14 | 19 | 23 | 16 | 17 |  |  |
| PDX | PDX1c_ACC_92/1 | ACC | X | Y | 17 | 18 | 13 | 15 | 11 | 14 | 13 | 16 | 8 | 11 | 12 | 13 | 12 | 13 | 12 | DO | 20 |  | 12 |  | 8 | 13 | 8 | 9.3 | 17 | 18 | 28 | 30 | 10 |  | 10 | 11 | 8 |  |  | 14 |  | 17 | 22 | 13 | 14 | 19 | 23 | 16 | 17 | Concordant | Dorp Out (DO): |
| Human | P_OS_111/1 | OS | X | Y | 15 | 18 | 16 | 17.3 | 11.3 | 14 | 14 | 16 | 11 | 12 | 12 | 15 | 9 |  | 16 | 18 | 19 | 25 | 10 | 12 | 10 | 11 | 6 | 8 | 15 | 18 | 29 | 32.2 | 7 | 12 | 12 | 14 | 6 | 8 | 10 | 15 | 16 | 18 | 20 | 12 | 15 | 19 | 24 | 15 |  |  |  |
| PDX | PDX2c1_OS_111/1 | OS | X | Y | 18 | DO | 16 | 17.3 | 11.3 | 14 | 14 | 16 | 11 | 12 | 15 | DO | 9 |  | 16 | 18 | 19 | 25 | 10 | 12 | 10 | 11 | 6 | 8 | 15 | DO | 29 | 32.2 | 7 | 12 | 12 | 14 | 6 | 8 | 10 | 15 | 16 | 20 | DO | 12 | 15 | 19 | 24 | 15 |  | Concordant | discrepancies: |
| Human | P_ACC_134/1 | ACC | X | Y | 16 |  | 14 | 17 | 11 |  | 16 | 17 | 8 | 12 | 8 | 16 | 12 |  |  | 19 |  | 18 | 11 | 13 | 9 |  | 6 |  | 16 | 17 |  | 31.2 | 8 | 10 | 12 | 13 | 9 |  | 10 | 13 | 14 | 18 | 19 | 12 |  | 23 | 24 | 15 | 17 |  |  |
| PDX | PDX2c3_ACC_134/1 | ACC | X | Y | 16 |  | 14 | 17 | 11 |  | 16 | 17 | 8 | 12 | 8 | 16 | 12 |  | 18 | 19 | 13 | 18 | 11 | 13 | 9 |  | 6 |  | 16 | 17 | 30.2 | 31.2 | 8 | 10 | 12 | 13 | 9 |  | 10 | 13 | 14 | 18 | 19 | 12 |  | 23 | 24 | 15 | 17 | Concordant | discrepancies: |
