# Supplementary material for: Establishing a pediatric solid tumor PDX biobank for precision oncology research
Source: Cancer Biol Ther. 2025 Aug 13;26(1):2541974. doi: 10.1080/15384047.2025.2541974 (PMC12351738; doi:10.1080/15384047.2025.2541974)
Supplement: Supplementary Methods.docx [file KCBT_A_2541974_SM8591.docx]

**Supplementary Methods**

**Flow cytometry analysis**

Xenograft tumor fragments (six 3 × 3 mm fragments) from PDX 40 were thawed at 37°C, washed with PBS and finely minced with a scalpel until complete dissociation. The minced tissue was then resuspended in 4 mL of an enzymatic solution composed of DMEM/F-12 medium supplemented with type II collagenase (275 U/mL) and incubated at 37°C, with gentle mixing every 5 minutes until detached cells suspension was observed. Enzymatic activity was halted by adding complete cell medium supplemented with 10% fetal bovine serum. The homogenate was filtered through a 100 µm cell strainer and centrifuged at 300 × g for 10 minutes. The resulting pellet was resuspended in PBS and cells were counted. To remove dead cells and debris, a Ficoll® density gradient centrifugation was performed at 13,000 × g for 5 minutes.

For flow cytometric analysis, 5 × 10³ cells from each single-cell suspension were used. Initially, cells were gated based on forward scatter (FSC) and side scatter (SSC) parameters to eliminate debris and non-viable cells. After that, analysis was restricted to the hCD45⁺ population using APC-anti-CD45 human (Biolegend, San Diego, CA, USA, #Cat 302330) and BV650-CD45 mouse (Biolegend, San Diego, CA, USA, #Cat 103139) which serves as a pan-lymphocyte marker. Within this gate, B and T lymphocyte subsets were identified using BV421-CD20 anti-human (Biolegend, San Diego, CA, USA, #Cat 302330), APC-Cy7 CD3 anti-human (Biolegend, San Diego, CA, USA, #Cat 300318), respectively. Flow cytometry was performed on an LSRFortessa™ X-20 (BD Biosciences, Franklin Lakes, NJ, USA), and data were acquired and analyzed using FlowJo v10.9 (Tree Star).
